# Combined effects of air pollution and meteorological factors on the risk of newly tuberculosis cases: a time-series study in Tibet, China

**DOI:** 10.1007/s00484-025-03032-0

**Published:** 2025-11-20

**Authors:** Luo Guo, Jingran Dong, Junfeng Zhang, Zhuang Cui, Changping Li, Su Wu

**Affiliations:** 1 Department of Industrial Engineering, Tsinghua University, Shunde Building South 611, Beijing, 100084 China; 2 Health and Health Committee of Tibet Autonomous Region, Tibet, 850000 China; 3 Department of Epidemiology and Biostatistics, School of Public Health, Tianjin Medical University, Tianjin, 300070 China

**Keywords:** Tuberculosis, Air pollution, Meteorological factors, High-altitude regions, PM_10_, Precipitation, Environmental health

## Abstract

The Tibet Autonomous Region, the world’s highest region, faces high tuberculosis (TB) rates, exacerbated by its unique environment and climate. However, the combined impact of air pollution and meteorological factors exposure on the prevalence of TB remains understudied. Daily data on TB cases, air pollutants (PM_10_, PM_2.5_, SO_2_, NO_2_, O_3_, and CO), and meteorological factors (temperature, precipitation, and wind speed) between 2019 and 2023 were collected. Weighted Quantile Sum (WQS) regression and Bayesian Kernel Machine Regression (BKMR) models were employed to assess the combined and individual effects of these environmental factors on TB prevalence. Moreover, gender, occupation, and year-specific distinction were explored using subgroup analysis. A total of 18,347 new TB cases were reported during the study period, with a positive association between environmental factors and the prevalence of TB. The WQS model showed a positive combined effect of environmental factors on daily TB cases (OR:1.58, 95%CI:1.46–1.71), and the weight of precipitation and PM_10_ were 0.55 and 0.59, respectively. BKMR analysis further confirmed a positive association between overall environmental factors and TB, highlighting precipitation as the most significant independent factor and potential interactions among environmental variables. Subgroup analyses confirmed a consistent positive association between environmental factors and TB cases, with PM_10_ and precipitation being most influential, precipitation more significant for females, and PM_10_ dominant among farmers and students. Exposure to air pollution and meteorological factors has a significant impact on the cases of TB. Notably, PM_10_ and precipitation are identified as primary determinants, with distinct variations observed among females, farmers, and students. Accurate estimates are essential for informing public health interventions, optimizing resource allocation, and developing effective clinical strategies in high-altitude regions.

**Supplementary Information:**

The online version contains supplementary material available at 10.1007/s00484-025-03032-0.

## Introduction

Tuberculosis (TB) is one of the leading causes of mortality, and pulmonary tuberculosis accounts for the vast majority of these deaths (Furin et al. 2019). According to a global survey by the World Health Organization (WHO), China is heavily burdened with TB and accounts for 6.8% of global TB patients (*Global Tuberculosis Report 2024* 2024). Although the incidence of TB has declined in recent years, it remains a major public health concern, due to its airborne transmission by *Mycobacterium tuberculosis* (MTB) (Koch and Mizrahi 2018; Vasiliu et al. 2024). High TB incidences will further hinder social and economic development, thus early detection and intervention of TB risk factors is essential to effectively reduce the burden of disease associated with TB (O’Donnell and Mathema 2022). Previous studies have shown that diabetes and HIV are the main risk factors (Bisht et al. 2023; Goletti et al. 2023; Schutz et al. 2010) for TB.

With the frequent occurrence of air pollution, the health effects of multi-pollutant mixtures are being paid (Nie et al. 2024; Xiong et al. 2022). Air pollution and meteorological conditions contribute significant to a range of health issues affecting multiple body systems. Recent studies have also indicated that air pollution and meteorological factors are key factors in the pathogenesis of several diseases, including cardiovascular disease (Kim et al. 2021; Rus and Mornoş 2022), COVID-19 (Mathys et al. 2023), allergic diseases (Hu et al. 2020; Luo et al. 2023), and autoimmune eye diseases (Cao et al. 2023). Growing attention has been paid to the effects of combined exposure to air pollutions and meteorological conditions on respiratory diseases, particularly TB, has attracted considerable interest in recent years. Some epidemiological studies have explored the relationship between exposure to air pollution and meteorological factors and the risk of TB, but the study findings have been inconsistent. Some studies (Dimala and Kadia 2022) showed that exposure to PM_2.5_, PM_10,_ and SO_2_ was found to be associated with an increased incidence of TB, while exposure to CO, NO_2,_ and O_3_ was not. However, a study in China, Urumqi showed that both pollutants and meteorological factors influence TB, and there may be an interaction between the effects on TB (Nie et al. 2024). Meteorological conditions, particularly ambient temperature (Chen et al. 2017), can modify the health risk of air pollution by influencing pollutant emissions dispersion, and concentrations (e.g.,PM_2.5_), while also acting as an independent risk factor for respiratory diseases (Kioumourtzoglou et al. 2016; Li et al. 2021). This inconsistency may stem from differences in geographical locations and the continuous development of economic levels. Therefore, understanding air pollution and meteorological factors in Tibet is crucial not only for the prevention and control of tuberculosis in the region but also for addressing the impacts of climate change in Asia and globally.

The annual incidence rate of TB in the Tibet Autonomous Region is much higher than the national average (Jiang et al. 2021) due to the relatively poor medical and health conditions in the region and the unbalanced distribution of health resources. Yet, high rates of TB in the Tibet Autonomous Region are likely linked to genetic adaptations to high altitude, which may dampen inflammatory responses to hypoxia (Corbett et al. 2022). Given these concerns, our study focused on the combined effects of multiple air pollutants and meteorological factors on TB, using data collected in Tibet from 2019 to 2023. We implemented a weighted Quantile Sum (WQS) and Bayesian Kernel Machine Regression (BKMR) approach to flexibly model the combined effects of exposure to air pollution and meteorological factors. The findings from this study are anticipated to suggest potential avenues for prevention and interventions on TB, thereby facilitating the reduction of future TB burdens.

## Materials and methods

### Study area

**Fig. 1:**
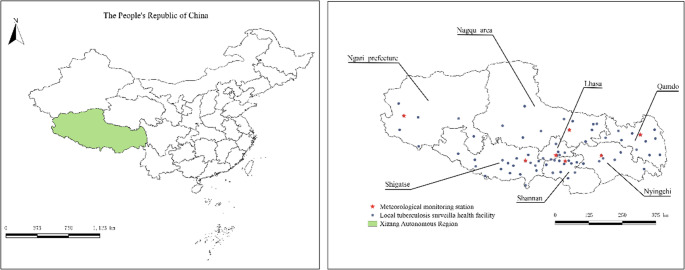
Distribution map of the local TB surveillance health facility and meteorological monitoring stations in Tibet Autonomous Region

Tibet Autonomous Region (78°25′−99°06′E, 26°50′−36°53′N) is a southwest area of China, is the “Roof of the World” of Tibetan, with an average altitude of more than 4,000 m. The region has a unique and complex climate due to the influence of its topography, geomorphology, and atmospheric circulation, which is characterized by cold and dry in the northwest, and warm and humid in southeast. Figure 1 shows the local TB surveillance health facility and main meteorological monitoring stations in different areas of Tibet.

### Tuberculosis data

Tuberculosis data used in this study were obtained from the routine surveillance system of the Tibet Center for Disease Control and Prevention (CDC), which were collected through the National Notifiable Disease Reporting System (NNDRS), a standardized, web-based platform maintained by the Chinese CDC. Key clinical and demographic data, together with regular quality checks to guarantee correctness and completeness, were recorded by qualified healthcare personnel and validated by experts. TB was diagnosed in accordance with national guidelines, based on clinical symptoms, chest radiographic findings, and laboratory tests, including sputum smear microscopy, culture, and molecular assays (Kontsevaya et al. 2024). Case definitions followed the Chinese National TB Diagnostic Criteria (WS 288–2017), including clinically diagnosed cases, suspected cases, and etiologically confirmed cases. Additional variables collected included age, sex, occupation, residential altitude, TB type, comorbidities, time to diagnosis, and treatment outcome. Our time-series study was based on daily reported TB case counts from 2019 to 2023, structured as a daily time series. Data covered all prefecture-level divisions within the Tibet Autonomous Region, including Lhasa, Shigatse, Shannan, Nyingchi, Qamdo, Nagqu, and Ngari region. However, our main analysis was based on province-level aggregated data. Further stratification in sparsely populated areas (e.g., only 455 TB cases were reported in Ngari from 2018 to 2023) would have resulted in limited statistical power.

### Exposure data

We collected daily data on outdoor air pollutants and meteorological factors between January 1, 2019, and December 31, 2023, extracted from the National Urban Air Quality Real-time Release Platform and the China Meteorological Data Sharing Center, respectively, which were widely utilized in previous studies (Chen et al. 2018; Liu et al. 2018). Air pollutants included PM_10_ (µg/m^3^), PM_2.5_ (µg/m^3^), SO_2_ (µg/m^3^), NO_2_ (µg/m^3^), O_3_ (ug/m^3^) and CO (mg/m^3^). Meteorological factors included average temperature (°C), maximum temperature (°C), minimum temperature (°C), wind speed (m/s) and precipitation (mm). Supplementary Table 1 compiled prefecture-level TB case counts, weather and air pollution indicators.

### Statistical analysis

The distribution attributes of TB, air pollutants, and meteorological factors were all subjected to descriptive statistical analysis, which metrics including mean ± standard deviation (SD), maximum value, minimum value, and various quantiles (P25, P50, P75). Correlations between daily concentrations of air pollutants and meteorological factors were analyzed by the Spearman rank correlation method to explore their relationship. In instances where the correlation coefficient exceeded 0.7, the variables were not incorporated into the same model as a control variable.

Then, WQS analysis was conducted to assess the multiple components effects of air pollutants, and meteorological factors on the number of TB cases by calculating a weighted Linear index and assigning corresponding weights. In our study, bootstrapping with 1,000 iterations was used to construct WQS indexes in both positive and negative directions, and when the WQS index showed significance, corresponding weights were examined to determine the relative contributions of various air pollutants and meteorological factors within the number of TB cases.

In addition, BKMR model was used to explore the overall effects of air pollutants, and meteorological factors on the number of TB cases. The model was involved 1,000 iterations to ensure model convergence and the influence of all factors on the number of TB cases was evaluated by calculating the posterior inclusion probability (PIP). By examining the impact of exposure levels in specific quartiles compared to the median, both univariate and bivariate exposure-response functions were employed to assess the single effect and interaction of all factors.

All analyses, including Spearman rank correlation, WQS, and BKMR were performed using the R software. *P* < 0.05 was statistically significant.

## Results

### Basic distributions

**Fig. 2:**
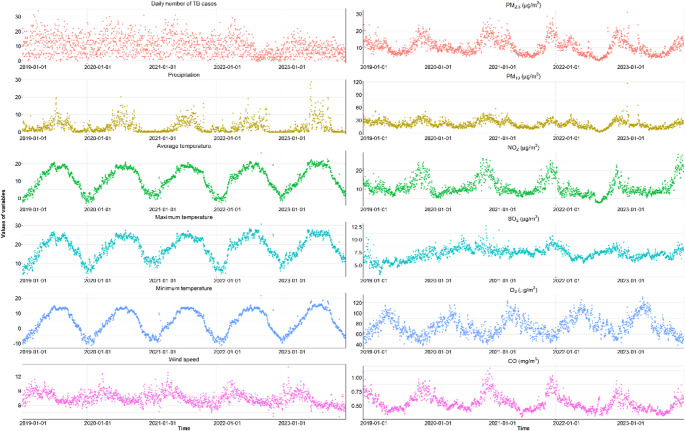
Temporal distribution of reported tuberculosis cases and meteorological factor indicators and air pollutants in Tibet Autonomous Region from 2019 to 2023

Table 1 presents the distribution of daily the number of TB cases, air pollutants, as well as meteorological factors. 18,347 new TB cases were recorded between January 1, 2019, and December 31, 2023, with a mean (± SD) of 10.05 ± 6.24 cases per day. 23.47% of cases were aetiologically proven, while 75.03% of cases were clinically diagnosed. Of the instances for whom bacteriological test results were available, 82.3% tested positive by molecular means and 7.8% tested positive by smear. The overall number of TB patients was 9707 (52.91%) men and 8640 (47.09%) females, with a male to female sex ratio of 1.12. Additionally, 14,389 (78.43%) were working age (15–60 years old), 2759 (15.04%) were > = 65 years old, and 1198 (6.53%) were under the age of 14. In the years immediately after 2021, the daily number of TB cases decreased significantly. Figure 2 shows the temporal variations of overall daily new TB cases, air pollutants and meteorological factors over the study period.

**Table 1:** Distribution characteristics of tuberculosis patients, air pollutants indicators, and meteorological factors in Tibet during 2019–2023

Variables	Daily number of cases *N* (%)	Mean ± SD	Min	Percentiles			Max
				25th	50th	75th	
Total	18,347 (100.00)	10.05 ± 6.24	0.00	5.00	9.00	14.00	34.00
Sex							
Male	9707 (52.91)	4.73 ± 3.41	0.00	2.00	4.00	7.00	18.00
Female	8640 (47.09)	5.32 ± 3.66	0.00	2.00	5.00	8.00	19.00
Age							
0 ~ 18	4567 (24.89)	2.50 ± 2.30	0.00	1.00	2.00	4.00	18.00
19 ~ 65	11,782 (64.22)	6.45 ± 4.25	0.00	3.00	6.00	9.00	23.00
>= 65	1997 (10.89)	1.09 ± 1.23	0.00	0.00	1.00	2.00	9.00
Work							
Nomads	11,908 (64.90)	6.52 (4.42)	0.00	3.00	6.00	9.00	25.00
Students	4458 (24.30)	2.44 (2.30)	0.00	1.00	2.00	4.00	19.00
other	1981 (10.80)	1.09 (1.17)	0.00	0.00	1.00	2.00	8.00
Year							
2019	4266 (23.25)	3.11 ± 6.68	0.00	6.00	12.00	16.00	34.00
2020	4377 (23.86)	11.96 ± 6.42	1.00	7.00	12.00	16.75	31.00
2021	4030 (21.97)	11.04 ± 6.11	0.00	6.00	11.00	15.00	31.00
2022	2865 (15.62)	7.85 ± 5.99	0.00	3.00	6.00	11.00	29.00
2023	2809 (15.31)	7.70 ± 4.26	0.00	5.00	7.00	11.00	22.00
Air pollutants indicators							
PM_10_ (µg/m^3^)		21.62 ± 8.85	2.43	15.33	20.52	26.77	116.29
PM_2.5_ (µg/m^3^)		10.59 ± 4.54	2.33	7.06	9.23	13.29	30.96
SO_2_ (µg/m^3^)		7.26 ± 1.15	3.22	6.51	7.30	7.99	12.68
NO_2_ (µg/m^3^)		11.01 ± 4.53	2.50	8.05	9.70	13.00	28.40
CO (mg/m^3^)		0.55 ± 0.14	0.30	0.45	0.52	0.62	1.16
O_3_ (µg/m^3^)		74.94 ± 18.10	37.87	61.21	72.30	87.31	130.86
Meteorological factors							
Average temperature (°C)		11.17 ± 6.63	−2.92	5.41	11.95	17.37	26.22
Maximum temperature (°C)		18.19 ± 5.93	4.22	13.46	18.97	23.44	30.59
Minimum temperature (°C)		4.23 ± 7.69	−11.45	−2.61	4.62	11.88	21.73
Wind speed (m/s)		7.48 ± 1.28	0.00	0.30	1.65	4.40	28.69
Precipitation (mm)		3.01 ± 3.65	3.63	6.57	7.37	8.27	14.01

The correlation coefficients of daily concentrations between air pollutants and meteorological factors were showed in Table 2 and supplementary Fig. 1. Among air pollutants indicators, PM_2.5_ was strongly correlated with PM_10_ (*r* = 0.89, *p* < 0.001) and CO (*r* = 0.77, *p* < 0.001), and CO was clearly negative correlation between temperature. While among meteorological factors, minimum temperature was strongly correlated with PM_2.5_ (*r* = − 0.77, *p* < 0.001), CO (*r* = − 0.78, *p* < 0.001), and precipitation (*r* = 0.71, *p* < 0.001). Considering the correlations for all other factors were weak and referring to relevant literature (Mao et al. 2023; Marx et al. 2021), we discarded CO, maximum and minimum temperature in following analyses.

**Table 2:** Correlation between daily air pollutant concentrations and meteorological factors

Variables	PM_10_	PM_2.5_	SO_2_	NO_2_	CO	O_3_	Tavg	Tmax	Tmin	Wspd	Prcp
PM_10_	1.00										
PM_2.5_	0.89***	1.00									
SO_2_	0.11***	0.23***	1.00								
NO_2_	0.65***	0.68***	0.29***	1.00							
CO	0.64***	0.77***	0.30***	0.61***	1.00						
O_3_	−0.32***	−0.37***	−0.31***	−0.64***	−0.49***	1.00					
Tavg	−0.58***	−0.73***	−0.22***	−0.45***	−0.78***	0.32***	1.00				
Tmax	−0.55***	−0.69***	−0.15***	−0.42***	−0.76***	0.30***	0.99***	1.00			
Tmin	−0.63***	−0.77***	−0.23***	−0.47***	−0.78***	0.30***	0.98***	0.98***	1.00		
Wspd	0.21***	0.11***	−0.26***	−0.32***	0.02	0.49***	−0.11***	−0.11***	−0.13***	1.00	
Prcp	−0.61***	−0.68***	−0.24***	−0.49***	−0.55***	0.29***	0.64***	0.58***	0.71***	−0.11	1.00

*p < 0.05; **p < 0.01; *** *p* < 0.001; Correlation coefficients greater than 0.7 represent covariance and are indicated in bold type

*Tavg* Average temperature, *Tmax* Maximum temperature, *Tmin* Minimum temperature, *Wspd* Wind speed, *Prcp* Precipitation

### WQS model

**Fig. 3:**
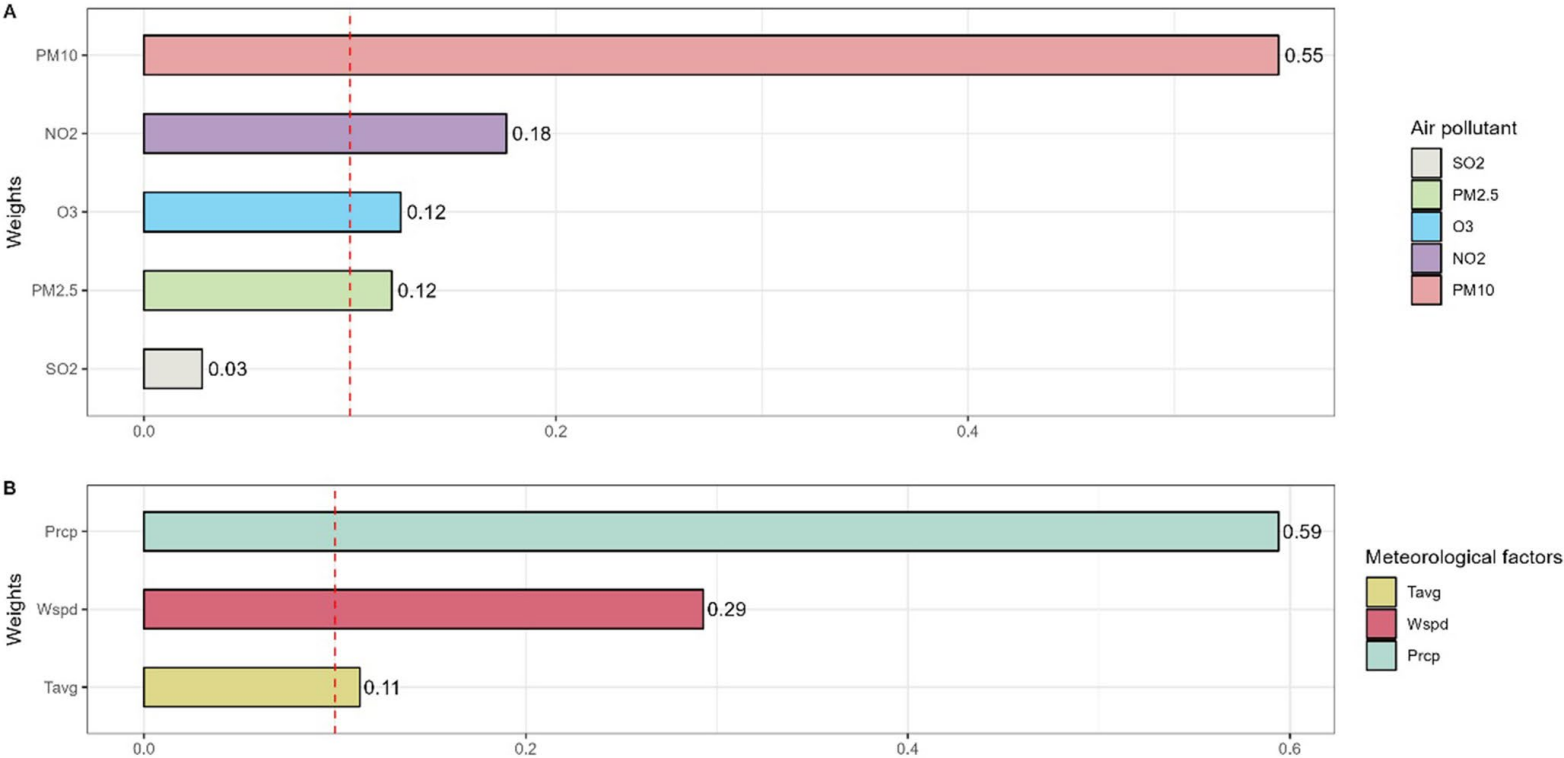
The WQS model weights of air pollution mixtures and meteorological factors on the number of tuberculosis cases in positive direction. The model was not adjusted for covariates. WQS: weighted quantile sum; Tavg: Average temperature; Tmax: Maximum temperature; Tmin: Minimum temperature; Wspd: Wind speed; Prcp: Precipitation

As presented in Table 3, the WQS model was applied to investigate the association between combined environmental exposures and the daily number of TB cases. In the crude model without covariate adjustment, we observed a strong positive association: each unit increase in the WQS index was associated with an 36.71% increase in the daily number of TB cases (OR = 1.58, 95% CI: 1.46–1.71). After adjusting for potential confounders, the associations remained statistically significant. In Model ^b^, which adjusted for all meteorological factors, the WQS index remained positively associated with TB cases (OR = 1.17, 95% CI 1.11–1.23, *P* < 0.001). Similarly, in Model ^d^, which adjusted for all air pollution factors, the association persisted (OR = 1.20, 95% CI 1.14–1.27, *P* < 0.001). Figure 3 shows the results of the WQS model weights of air pollution mixtures and meteorological factors on the number of TB cases in positive direction. Specifically, precipitation had the largest contribution to the increased risk of TB cases (weight: 0.55) and wind speed had the second largest contribution (weight: 0.29). The results of WQS model weights among air pollution mixtures on the number of tuberculosis cases, PM_10_ had the largest contribution (weight: 0.59). The WQS regression in the negative direction did not show significant association of the environment mixtures with the number of TB cases, as shown in Supplementary Table 2.

**Table 3:** Combined effect of air pollution mixtures and meteorological factors on the number of tuberculosis cases in the WQS model

Outcome	OR	95% CI	*P*-value
Mix model	1.58	1.46–1.71	**< 0.001**
Air pollutants indicators			
Crude model ^a^	1.22	1.16–1.28	**< 0.001**
Model ^b^	1.17	1.11–1.23	**< 0.001**
Meteorological factors			
Crude model ^c^	1.20	1.15–1.25	**< 0.001**
Model ^d^	1.20	1.14–1.27	**< 0.001**

*WQS* weighted quantile sum, *OR* odds ratio, *CI* confidence interval, *Tavg* Average temperature, *Tmax* Maximum temperature, *Tmin* Minimum temperature, Wspd Wind speed Prcp: Precipitation

Crude model (a, c): No covariates were adjusted

Model b: Adjusted for all meteorological factor

Model d: Adjusted for all air pollution factors

### BKMR model

**Fig. 4:**
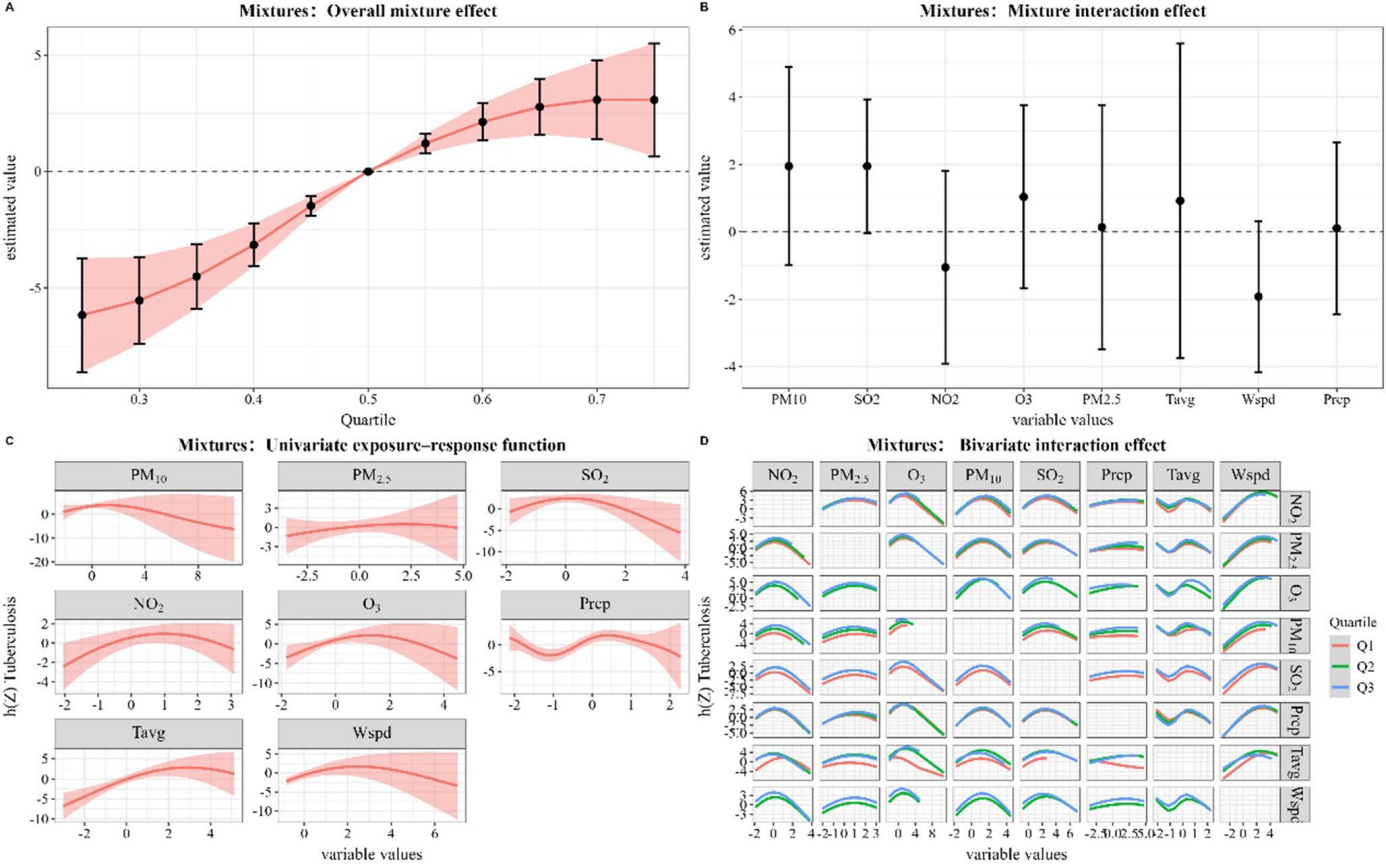
Overall effect of environment mixtures on the number of tuberculosis cases in BKMR model. All factors at specific percentiles were compared to their 50th percentile. The model adjusted for all covariates. BKMR: Bayesian kernel machine regression. **A**: The mixed overall effect of exposure to air pollutants and meteorological factors on the number of tuberculosis cases. **B**: Mixed interactive effects of mixed exposure to air pollutants and meteorological factors on the number of tuberculosis cases. **C**: Univariate dose-response relationship diagram of mixed exposure to air pollutants and meteorological factors on tuberculosis. **D**: Bivariate dose-response relationship diagram of mixed exposure to air pollutants and meteorological factors on tuberculosis

In the BKMR model, we assessed the joint and individual effects of environmental mixtures on the daily number of TB cases. As summarized in supplementary Table 3, all environment mixtures were identified as key contributors, with relatively high PIPs. Figure 4 A illustrates the overall effect of environment mixtures when all factors were simultaneously fixed at specific percentiles, a significant and approximately linear positive association was observed between the mixture index and the number of TB cases. As shown in Fig. 4B, when considering all mixtures, none of the interaction effects of the single environmental factor in this mixture. Figure 4 C present a significant linear positive correlation between the average temperature and the number of TB cases in univariate exposure-exposure. However, precipitation showed a significant non-linear positive correlation with the number of TB cases, and none of the other factors showed a statistically significant relationship with the number of TB cases. Figure 4D shows the effect of all environmental factors on the number of TB cases, fixing the study factors at their respective Q1, Q2 and Q3, the results showed that for PM_10_, O_3_, average temperature and other factors, the exposure-response curves were found to have a non-parallel trend through curve visualization, indicating a potential interaction between the environmental factors. Unfortunately, the BKMR model did not include adjustments for additional demographic or socioeconomic confounders due to data limitations.

### Subgroup analysis and sensitivity analysis

Subgroup analysis was conducted to assess the consistency of results, and the positive association between all environment factors and the number of TB cases remained consistent in subgroups stratified by gender, occupation and year. In the WQS model, precipitation was significantly more important for females compared to males, while the importance of other variables, such as PM2.5, average temperature, and SO2, decreased notably. Additionally, the weight of NO2 was higher for females than males, showing a significant increase (Supplementary Figs. 5–6). The BKMR model further demonstrated a significant positive effect of environmental mixtures on TB cases in both genders (Supplementary Figs. 15–16). By work group, among farmers and students, the weight of PM_10_ has been dominant, while SO_2_ and precipitation hold a more important position in other occupations in the WQS model (Supplementary Figs. 7–9), and precipitation was still the main factors affecting the number of TB cases in the BKMR model (Supplementary Figs. 17–19). Furthermore, we divide the data into two parts by year, between the year of 2019 to 2021, the weight of precipitation has been dominant, while wind speed hold a more important position between 2022 and 2023 in the WQS model (Supplementary Figs. 10–11), while PM_2.5_ become the main factors affecting the number of TB cases in the BKMR model (Supplementary Figs. 20–21). Subgroup analyses, though revealing subtle variations, consistently identified PM_10_ and precipitation as the most influential factors, whether considered individually or in combination. In addition, we performed a sensitivity analysis based on monthly data, accounting for time trends to further assess the impact of environmental exposures on tuberculosis incidence. As part of the sensitivity analyses, we applied both models (Supplementary Fig. 14 and Supplementary Fig. 24) in a region-stratified manner. The consistent identification of PM₁₀ as a major risk factor and the reproducibility of region-specific precipitation effects confirm the robustness of our findings across both models and geographic strata. Considering the incubation period of TB, we employed monthly aggregated data — where TB cases were summed by month and meteorological as well as air pollution factors were averaged — to complement our main analyses and more comprehensively capture broader environmental influences on TB. Although no formal lag analysis was performed, the results from these supplementary analyses were consistent with those of the primary daily-level analysis, further supporting the robustness of our findings (see Supplementary Figs. 12 and 22). By applying natural spline functions to flexibly control for long-term and seasonal time trends in the time-series analysis, Supplementary Figs. 13 and 23 produced results consistent with the primary analysis, reinforcing the reliability of the identified associations between PM₁₀, precipitation, and tuberculosis incidence. For further details, please refer to the supplementary file.

## Discussion

This study utilized WQS and BKMR models to assess the impact of air pollution and meteorological factors on the daily number of TB cases from a time-series perspective. The findings demonstrate that combined exposure to air pollutants and meteorological factors is significantly and positively associated with an increased risk of daily TB cases, with PM_10_ and precipitation contributing most prominently. Furthermore, significant interactions were identified between PM_10_, average temperature, and the risk of TB cases. Notably, this is the first time-series analysis to investigate the impact of combined environmental exposure on TB risk in a high-altitude plateau region of China. Subgroup analyses examining differences across genders, occupations, and years further validated the consistency of the results.

Our study identified a significant association between PM_10_ exposure and the risk of new TB cases. Previous studies have emphasized that short-term exposure to environmental air pollutants, especially PM_2.5_ (Wang et al. 2023) will significantly increase the risk of TB, with studies showing a 12% increase in TB incidence for every 10 µg/m³ rise in annual PM_2.5_ levels (Li et al. 2019). However, current evidence linking PM_10_ exposure to TB risk remains inconclusive, some epidemiological studies, which have not reported a significant link between PM_10_ and TB (Hwang et al. 2014; Liu et al. 2020; Smith et al. 2014; Smith et al. 2016). A case-crossover study conducted in Hainan, China, found that while PM_10_ levels were positively associated with TB risk, the observed effects were notably weaker than those linked to PM_2.5_ exposure (Zhu et al. 2023). The mucous membranes in the respiratory tract protect against MTB, but coarse particulate air pollution (Feng et al. 2024) can compromise this defense, allowing pathogens to reach the alveoli and cause pulmonary diseases (Ni et al. 2015; Zhu et al. 2018). Therefore, our study focused on linking PM_10_ exposure with adverse respiratory effects, with an emphasis on potential risks to TB. Previous experimental studies have shown that PM_10_ can promote intracellular growth of MTB by inducing cellular senescence and suppressing the expression of antimicrobial peptides, human β-defensin 2 (HBD-2) and human β-defensin 3 (HBD-3), both of which play crucial roles in the early defense against TB infection (Rivas-Santiago et al. 2006, 2015). Further research is needed to better understand the potential mechanisms and associations between PM10 exposure and TB incidence.

The link between climate and TB has been recognized for thousands of years (Jones Jr 1954), with studies showing that weather changes contribute to the mechanisms underlying respiratory disease development (Josa-Culleré et al. 2024; Motlogeloa and Fitchett 2024; Wu et al. 2024). Our study highlights that precipitation and average temperature may influence the daily number of TB cases. Precipitation is a key meteorological factor influencing TB, and our analysis revealed it had the most significant impact among all factors, showing a positive correlation with TB, which was consistent with the results of the study in a systematic review and meta-analysis (Qin et al. 2022). Weather factors influence TB transmission by affecting MTB survival, human behavior, and susceptibility. Precipitation reduces sunlight (Liu et al. 2012) and ultraviolet radiation (Miller 2013), which promotes antimicrobial activity by inhibiting inflammasome activation and cell death, while also regulating vitamin D production, which enhances antimicrobial and anti-inflammatory effects (Abhimanyu and Coussens 2017). Furthermore, precipitation increases indoor time, which in turn boosts MTB transmission by promoting person-to-person contact, crowding, and poor ventilation (Naranbat et al. 2009; Yang et al. 2016). Unlike precipitation, the correlation between temperature and TB is not clear significant (Chang et al. 2024), while MTB thrives in moderate temperatures (Farida et al. 2013), both cold and extreme heat can reduce human metabolic functions (Maharjan et al. 2021). Temperature can affect the activities of susceptible people, and more people engage in outdoor activities when the temperature is within the optimal range. As previously stated, high precipitation and average temperature are favorable for MTB growth, thus, the effects of meteorological factors on TB cases should be focused.

Notably, the sustained decline in TB incidence observed after 2021 may reflect the combined impact of multiple targeted interventions in Tibet. These include the large-scale replacement of biomass fuels, upgraded mobile screening strategies, and improved treatment protocols under hypoxic conditions. However, ongoing monitoring is essential to distinguish the true long-term trend from short-term fluctuations influenced by the COVID-19 pandemic response. Despite the studies (Dimala et al. 2020; Tang et al. 2023) explored the relationship between high-altitude environments, air pollution, meteorological factors, and TB risk, but high altitude areas research remains limited, such as Tibet. Although studies showing that higher altitudes generally have better air quality, with lower pollutant concentrations and improved visibility, due to enhanced atmospheric mixing, reduced human activity, and meteorological factors like wind speed and humidity (Ji et al. 2018). However, the higher annual TB incidence in the Tibet Autonomous Region, driven by limited healthcare and resource imbalance, may also be linked to genetic adaptations to high altitude that affect inflammatory responses to hypoxia (Corbett et al. 2022; Jiang et al. 2021). Although Tibet is known for its clean outdoor air, indoor air pollution poses a significant but overlooked health risk. In rural areas, burning biomass fuels like cow dung and firewood in poorly ventilated homes releases harmful pollutants, impairing respiratory defenses and increasing the risk of TB progression. High altitude further exacerbates this vulnerability: chronic hypoxia weakens immune function, and the cold climate leads to prolonged indoor stays in crowded settings with limited ventilation, facilitating TB transmission. Together, these environmental and physiological factors create a high-risk context for TB in the plateau population. Seasonal variation may also contribute to the observed patterns in TB incidence (Chong et al. 2022). During colder months, individuals in Tibet are more likely to remain indoors in poorly ventilated settings and rely on biomass fuels for heating, which increases exposure to indoor air pollutants. Moreover, seasonal fluctuations in temperature, humidity, and wind patterns can influence the dispersion and concentration of outdoor air pollutants, potentially modifying respiratory vulnerability and TB transmission risks. Hence, consideration of seasonal effects is imperative in the context of this study. Due to air pollutants and meteorological factors are global concerns, as their interaction, particularly during extreme weather events like heatwaves, significantly increases respiratory and cardiovascular risks, especially among vulnerable groups such as the elderly and children (Cheng et al. 2024; Weichenthal et al. 2014). Therefore, understanding these interactions are particularly critical in high-altitude regions like Tibet, where the combined effects of air pollutants and unique climatic factors on diseases such as TB remain underexplored.

Our study offers several notable strengths. First, it employed both WQS and BKMR models to comprehensively evaluate the impact of air pollution and meteorological factors on TB from multiple perspectives. Covering daily TB case data and environmental metrics in the Tibet from 2019 to 2023, this research spans a significant timeframe and focuses on a uniquely high-altitude region. To our knowledge, it is the first to systematically explore the role of short-term environmental exposure in plateau regions on TB risk. Additionally, subgroup analyses confirmed the robustness of our findings, providing a solid scientific basis for developing TB prevention strategies tailored to environmental and meteorological conditions in plateau areas, thereby addressing a critical research gap in this field.

Admittedly, this study has a few limitations. First, the study is limited to Tibet and highlights a potential link between precipitation, temperature, and TB, but further research and experimental validation are needed to confirm its biological mechanisms and broader applicability. Additionally, although some confounding factors were controlled, other influential factors, such as economic status and access to healthcare, were not fully incorporated into the analysis, which may affect the precision and robustness of the conclusions. Additionally, the time-series approach employed in our study might not fully capture causal correlations based on short-term exposure changes due to the lengthy latency period of TB. Our future research will completely take into account the development of underlying disorders and long-term exposure, since we recognise that our study does not accurately reflect the cumulative or long-term environmental impacts on the development of TB.

## Conclusion

In summary, our study demonstrates the significant combined impact of air pollution and meteorological factors on TB cases in the Tibet, with PM10 and precipitation identified as key contributors. These findings underscore the need for targeted public health measures to mitigate exposure, especially in high-altitude areas with unique climates. To better understand the multifactorial nature of TB risk, our future research will take more thorough approach, including individual-level exposure data, genetic susceptibility, and socioeconomic background, as well as long-term environmental consequences on TB development.

## Supplementary Information

Below is the link to the electronic supplementary material.

Supplementary Material 1

## Data Availability

The datasets generated and analyzed during the current study are not publicly available due to privacy restrictions, and institutional policies, but are available from the corresponding author upon reasonable request.

## Abbreviations

TB: Tuberculosis
WQS: Weighted Quantile Sum
BKMR: Bayesian Kernel Machine Regression
MTB: Mycobacterium tuberculosis

## Code availability

Custom code used in this study can be provided upon request from the corresponding author.
