# Supplementary material for: Combined effects of air pollution and meteorological factors on the risk of newly tuberculosis cases: a time-series study in Tibet, China
Source: Int J Biometeorol. 2025 Nov 20;69(12):3451–62. doi: 10.1007/s00484-025-03032-0 (PMC12689801; doi:10.1007/s00484-025-03032-0)
Supplement: Supplementary file 1 — Supplementary Material 1 [file 484_2025_3032_MOESM1_ESM.docx]

**Supplementary file contents**

[Supplementary Table 1 Summary statistics of the city-specific characteristics for the 7 prefecture-level regions in Tibet Autonomous Region, China. 3](#_Toc204346147)

[Supplementary Table 2 Combined effect of air pollution mixtures and meteorological factors on the number of tuberculosis cases in the WQS model 4](#_Toc204346148)

[Supplementary Table 3 PIPs of air pollution mixtures and meteorological factors for the number of tuberculosis cases in BKMR model. 5](#_Toc204346149)

[Supplementary Table 4 Combined effect of air pollution mixtures and meteorological factors on the number of tuberculosis cases in the WQS model *(Sex) 6](#_Toc204346150)

[Supplementary Table 5 Combined effect of air pollution mixtures and meteorological factors on the number of tuberculosis cases in the WQS model *(Work) 7](#_Toc204346151)

[Supplementary Table 6 Combined effect of air pollution mixtures and meteorological factors on the number of tuberculosis cases in the WQS model *(Year) 8](#_Toc204346152)

[Supplementary Table 7 PIPs of air pollution mixtures and meteorological factors for the number of tuberculosis cases in BKMR model. 9](#_Toc204346153)

[Supplementary Figure 1 Correlation between daily air pollutant concentrations and meteorological variables 10](#_Toc204346154)

[Supplementary Figure 2 The WQS model weights of air pollution mixtures and meteorological factors on the number of tuberculosis cases in positive direction. The model was adjusted for all covariates. 11](#_Toc204346155)

[Supplementary Figure 3 The BKMR model for joint effect analysis of meteorological factors on the number of tuberculosis cases. The model adjusted for all covariates. 12](#_Toc204346156)

[Supplementary Figure 4 The BKMR model for joint effect analysis of air pollutants on the number of tuberculosis cases. The model adjusted for all covariates. 13](#_Toc204346157)

[Supplementary Figure 5 The WQS model weights of the effects of air pollution mixture and meteorological factors on the number of tuberculosis cases in the male subgroup are positive. The model A and B were adjusted for all covariates and the model C was not adjusted for covariates. 14](#_Toc204346158)

[Supplementary Figure 6 The WQS model weights of the effects of air pollution mixture and meteorological factors on the number of tuberculosis cases in the female subgroup are positive. The model A and B were adjusted for all covariates and the model C was not adjusted for covariates. 15](#_Toc204346159)

[Supplementary Figure 7 The WQS model weights of the effects of air pollution mixture and meteorological factors on the number of tuberculosis cases in the nomad subgroup are positive. The model A and B were adjusted for all covariates and the model C was not adjusted for covariates. 16](#_Toc204346160)

[Supplementary Figure 8 The WQS model weights of the effects of air pollution mixture and meteorological factors on the number of tuberculosis cases in the student subgroup are positive. The model A and B were adjusted for all covariates and the model C was not adjusted for covariates. 17](#_Toc204346161)

[Supplementary Figure 9 The WQS model weights of the effects of air pollution mixture and meteorological factors on the number of tuberculosis cases in the other occupations subgroup are positive. The model A and B were adjusted for all covariates and the model C was not adjusted for covariates. 18](#_Toc204346162)

[Supplementary Figure 10 The WQS model weights of the effects of air pollution mixture and meteorological factors on the number of tuberculosis cases between year of 2019 and 2021 subgroup are positive. The model A and B were adjusted for all covariates and the model C was not adjusted for covariates. 19](#_Toc204346163)

[Supplementary Figure 11 The WQS model weights of the effects of air pollution mixture and meteorological factors on the number of tuberculosis cases between year of 2022 and 2023 subgroup are positive. The model A and B were adjusted for all covariates and the model C was not adjusted for covariates. 20](#_Toc204346164)

[Supplementary Figure 12 The WQS model weights of the effects of air pollution mixture and meteorological factors on the number of tuberculosis cases by monthly. The model A and B were adjusted for all covariates and the model C was not adjusted for covariates. 21](#_Toc204346165)

[Supplementary Figure 13 The WQS model weights of the effects of air pollution mixture and meteorological factors on the number of tuberculosis cases when adjusting for time trends. The model A and B were adjusted for all covariates and the model C was just adjusted for time trends. 22](#_Toc204346166)

[Supplementary Figure 14 The WQS model weights of air pollution mixtures and meteorological factors on the number of tuberculosis cases in positive direction (With different regions) 23](#_Toc204346167)

[Supplementary Figure 15 The BKMR model for joint effect analysis of air pollutants on the number of tuberculosis cases in the male subgroup. The model was not adjusted for covariates. 24](#_Toc204346168)

[Supplementary Figure 16 The BKMR model for joint effect analysis of air pollutants on the number of tuberculosis cases in the female subgroup. The model was not adjusted for covariates. 25](#_Toc204346169)

[Supplementary Figure 17 The BKMR model for joint effect analysis of air pollutants on the number of tuberculosis cases in the nomad subgroup. The model was not adjusted for covariates. 26](#_Toc204346170)

[Supplementary Figure 18 The BKMR model for joint effect analysis of air pollutants on the number of tuberculosis cases in the student subgroup. The model was not adjusted for covariates. 27](#_Toc204346171)

[Supplementary Figure 19 The BKMR model for joint effect analysis of air pollutants on the number of tuberculosis cases in the other occupation subgroup. The model was not adjusted for covariates. 28](#_Toc204346172)

[Supplementary Figure 20 The BKMR model for joint effect analysis of air pollutants on the number of tuberculosis cases between the year of 2019 to 2021. The model was not adjusted for covariates. 29](#_Toc204346173)

[Supplementary Figure 21 The BKMR model for joint effect analysis of air pollutants on the number of tuberculosis cases between the year of 2022 to 2023. The model was not adjusted for covariates. 30](#_Toc204346174)

[Supplementary Figure 22 The BKMR model for joint effect analysis of air pollutants on the number of tuberculosis cases by monthly. 31](#_Toc204346175)

[Supplementary Figure 23 The BKMR model for joint effect analysis of air pollutants on the number of tuberculosis cases when adjusting for time trends. 32](#_Toc204346176)

[Supplementary Figure 24 Overall effect of environment mixtures on the number of tuberculosis cases in BKMR model (With different regions). 33](#_Toc204346177)

# Supplementary Table 1 Summary statistics of the city-specific characteristics for the 7 prefecture-level regions in Tibet Autonomous Region, China.

| **Regions** | TB cases, N (%) | Air pollutants indicators, Mean ± SD | | | | | | Meteorological factors, Mean ± SD | | | | |
| --- | --- | --- | --- | --- | --- | --- | --- | --- | --- | --- | --- | --- |
|  |  | PM_10_ | PM_2.5_ | SO_2_ | NO_2_ | CO | O_3_ | Tavg | Tmax | Tmin | Wspd | Prcp |
| Total | 18347 (100.00) | 21.62 ± 8.85 | 10.59 ± 4.54 | 7.26 ± 1.15 | 11.01 ± 4.53 | 0.55 ± 0.14 | 74.94 ± 18.10 | 11.17 ± 6.63 | 18.19 ± 5.93 | 4.23 ± 7.69 | 7.48 ± 1.28 | 3.01 ± 3.65 |
| Lhasa | 1453 (7.92) | 19.61 ± 13.35 | 8.24 ± 5.63 | 6.08 ± 2.79 | 9.71 ± 4.61 | 0.43 ± 0.12 | 78.00 ± 21.49 | 8.19 ± 7.00 | 15.62 ± 6.38 | 1.20 ± 7.99 | 8.38 ± 1.72 | 2.05 ± 3.37 |
| Shigatse | 3772 (20.56) | 13.00 ± 6.97 | 6.24 ± 2.91 | 9.46 ± 4.10 | 10.72 ± 6.28 | 0.43 ± 0.14 | 90.79 ± 26.53 | 13.64 ± 6.13 | 20.19 ± 5.25 | 6.70 ± 7.44 | 7.32 ± 1.52 | 3.48 ± 5.35 |
| Shannan | 1558 (8.49) | 12.79 ± 7.36 | 7.52 ± 4.49 | 7.70 ± 2.19 | 9.71 ± 4.61 | 0.48 ± 0.18 | 67.42 ± 22.06 | 15.30 ± 5.87 | 21.74 ± 4.85 | 8.39 ± 7.23 | 6.51 ± 1.37 | 4.24 ± 5.85 |
| Nyingchi | 1360 (7.41) | 23.24 ± 13.94 | 10.42 ± 7.16 | 4.61 ± 1.73 | 11.15 ± 6.66 | 0.56 ± 0.20 | 80.31 ± 24.90 | 14.75 ± 5.59 | 20.98 ± 5.11 | 8.70 ± 6.57 | 6.68 ± 1.40 | 4.88 ± 7.48 |
| Chamdo | 6704 (36.54) | 20.84 ± 13.23 | 10.63 ± 6.67 | 8.33 ± 2.79 | 11.59 ± 7.56 | 0.44 ± 0.21 | 62.35 ± 20.61 | 8.31 ± 6.86 | 16.68 ± 6.53 | 1.23 ± 7.83 | 6.41 ± 1.13 | 2.22 ± 3.07 |
| Nagqu | 3044 (16.59) | 41.80 ± 20.87 | 23.02 ± 13.55 | 7.64 ± 3.05 | 10.95 ± 5.48 | 0.85 ± 0.39 | 66.60 ± 21.89 | 4.81 ± 8.03 | 12.30 ± 7.48 | -2.56 ± 9.12 | 10.16 ± 2.54 | 1.94 ± 2.85 |
| Ngari | 455 (2.48) | 6.25 ± 2.91 | 21.40 ± 13.29 | 7.07 ± 2.08 | 17.71 ± 9.74 | 0.44 ± 0.21 | 78.62 ± 24.05 | 13.17 ± 7.70 | 19.86 ± 7.11 | 5.95 ± 8.29 | 6.90 ± 1.49 | 2.23 ± 3.80 |
| Tavg: Average temperature; Tmax: Max temperature; Tmin: Mini temperature; Wspd: Wind speed; Prcp: Precipitation;  Note: Units – Temperature: °C; Wind speed: m/s; Precipitation: mm;  Air pollutants: PM₁₀, PM₂.₅, SO₂, NO₂, O₃ in μg/m³; CO in mg/m³. | | | | | | | | | | | | |

# Supplementary Table 2 Combined effect of air pollution mixtures and meteorological factors on the number of tuberculosis cases in the WQS model

| Outcome | OR | 95% CI | *P*-value |
| --- | --- | --- | --- |
| Mix model | 0.94 | 0.91-0.97 | **< 0.001** |
| Air pollutant mixtures | | | |
| Crude model I | 0.94 | 0.91-0.97 | **< 0.001** |
| Model I | 1.00 | 0.96-1.05 | 0.86 |
| Meteorological factors | | | |
| Crude model II | 1.03 | 1.00-1.06 | 0.09 |
| Model II | 1.19 | 1.13-1.27 | **< 0.001** |
| WQS: weighted quantile sum; OR: odds ratio; CI: confidence interval; Crude model adjusted for none, while Model I adjusted for all meteorological factor and Model II adjusted for all air pollution factors. | | | |

# **Supplementary Table 3** PIPs of air pollution mixtures and meteorological factors for the number of tuberculosis cases in BKMR model.

|  | PIP ^a^ | PIP ^b^ |
| --- | --- | --- |
| Air pollutant mixtures |  |  |
| PM_10_ (μg/m3) | 1.00 | 1.00 |
| NO_2_ (μg/m3) | 1.00 | 1.00 |
| SO_2_ (μg/m3) | 1.00 | 1.00 |
| O_3_ (μg/m3) | 1.00 | 0.91 |
| Meteorological factors |  |  |
| Average temperature (°C) | 1.00 | 1.00 |
| Wind speed (m/s) | 1.00 | 1.00 |
| Precipitation (mm) | 0.96 | 1.00 |
| ^a^ Exposure to air pollution mixtures alone or exposure to meteorological factors alone；This model adjusted for the covariates.  ^b^ Mixed exposure to air pollution mixtures and meteorological factors; The model was not adjusted for covariates. | | |

# Supplementary Table 4 Combined effect of air pollution mixtures and meteorological factors on the number of tuberculosis cases in the WQS model *(Sex)

| Outcome | OR | 95% CI | *P*-value |
| --- | --- | --- | --- |
| **Male** |  |  |  |
| Mix model | 1.67 | 1.51-1.85 | **< 0.001** |
| Air pollutants indicators | | | |
| Crude model I | 1.24 | 1.17-1.32 | **< 0.001** |
| Model I | 1.18 | 1.11-1.26 | **< 0.001** |
| Meteorological factors | | | |
| Crude model II | 1.21 | 1.15-1.27 | **< 0.001** |
| Model II | 1.27 | 1.18-1.36 | **< 0.001** |
| **Female** | | | |
| Mix model | 1.53 | 1.40-1.66 | **< 0.001** |
| Air pollutants indicators | | | |
| Crude model I | 1.20 | 1.15-1.26 | **< 0.001** |
| Model I | 1.17 | 1.11-1.24 | **< 0.001** |
| Meteorological factors | | | |
| Crude model II | 1.20 | 1.15-1.26 | **< 0.001** |
| Model II | 1.16 | 1.10-1.22 | **< 0.001** |
| WQS: weighted quantile sum; OR: odds ratio; CI: confidence interval; Crude model adjusted for none, while Model I adjusted for all meteorological factor and Model II adjusted for all air pollution factors.  Tavg: Average temperature; Tmax: Maximum temperature; Tmin: Minimum temperature; Wspd: Wind speed; Prcp: Precipitation | | | |

# Supplementary Table 5 Combined effect of air pollution mixtures and meteorological factors on the number of tuberculosis cases in the WQS model *(Work)

| Outcome | OR | 95% CI | *P*-value |
| --- | --- | --- | --- |
| **Nomads** |  |  |  |
| Mix model | 1.53 | 1.40-1.61 | **< 0.001** |
| Air pollutants indicators | | | |
| Crude model I | 1.19 | 1.13-1.26 | **< 0.001** |
| Model I | 1.18 | 1.12-1.25 | **< 0.001** |
| Meteorological factors | | | |
| Crude model II | 1.20 | 1.14-1.25 | **< 0.001** |
| Model II | 1.15 | 1.09-1.21 | **< 0.001** |
| **Students** |  |  |  |
| Mix model | 1.86 | 1.64-2.11 | **< 0.001** |
| Air pollutants indicators | | | |
| Crude model I | 1.32 | 1.22-1.42 | **< 0.001** |
| Model I | 1.31 | 1.21-1.41 | **< 0.001** |
| Meteorological factors | | | |
| Crude model II | 1.21 | 1.14-1.29 | **< 0.001** |
| Model II | 1.38 | 1.25-1.51 | **< 0.001** |
| **Other** | | | |
| Mix model | 1.48 | 1.27-1.73 | **< 0.001** |
| Air pollutants indicators | | | |
| Crude model I | 1.26 | 1.14-1.39 | **< 0.001** |
| Model I | 1.16 | 1.02-1.31 | **< 0.001** |
| Meteorological factors | | | |
| Crude model II | 1.18 | 1.09-1.28 | **< 0.001** |
| Model II | 1.19 | 1.08-1.31 | **< 0.001** |
| WQS: weighted quantile sum; OR: odds ratio; CI: confidence interval; Crude model adjusted for none, while Model I adjusted for all meteorological factor and Model II adjusted for all air pollution factors.  Tavg: Average temperature; Tmax: Maximum temperature; Tmin: Minimum temperature; Wspd: Wind speed; Prcp: Precipitation | | | |

# Supplementary Table 6 Combined effect of air pollution mixtures and meteorological factors on the number of tuberculosis cases in the WQS model *(Year)

| Outcome | OR | 95% CI | *P*-value |
| --- | --- | --- | --- |
| **2019-2021** |  |  |  |
| Mix model | 1.32 | 1.17-1.48 | **< 0.001** |
| Air pollutants indicators | | | |
| Crude model I | 1.16 | 1.08-1.24 | **< 0.001** |
| Model I | 1.15 | 1.07-1.25 | **< 0.001** |
| Meteorological factors | | | |
| Crude model II | 1.10 | 1.05-1.16 | **< 0.001** |
| Model II | 1.05 | 1.00-1.10 | 0.06 |
| **2022-2023** | | | |
| Mix model | 1.50 | 1.38-1.63 | **< 0.001** |
| Air pollutants indicators | | | |
| Crude model I | 1.37 | 1.27-1.48 | **< 0.001** |
| Model I | 1.31 | 1.18-1.45 | **< 0.001** |
| Meteorological factors | | | |
| Crude model II | 1.22 | 1.14-1.30 | **< 0.001** |
| Model II | 1.15 | 1.03-1.28 | **< 0.001** |
| WQS: weighted quantile sum; OR: odds ratio; CI: confidence interval; Crude model adjusted for none, while Model I adjusted for all meteorological factor and Model II adjusted for all air pollution factors.  Tavg: Average temperature; Tmax: Maximum temperature; Tmin: Minimum temperature; Wspd: Wind speed; Prcp: Precipitation | | | |

# Supplementary Table 7 PIPs of air pollution mixtures and meteorological factors for the number of tuberculosis cases in BKMR model.

|  | PIP ^a^ | PIP ^b^ |
| --- | --- | --- |
| Air pollutant mixtures |  |  |
| PM_10_ (μg/m3) | 1.00 | 1.00 |
| NO_2_ (μg/m3) | 1.00 | 1.00 |
| SO_2_ (μg/m3) | 1.00 | 1.00 |
| O_3_ (μg/m3) | 1.00 | 0.91 |
| Meteorological factors |  |  |
| Average temperature (°C) | 1.00 | 1.00 |
| Wind speed (m/s) | 1.00 | 1.00 |
| Precipitation (mm) | 0.96 | 1.00 |
| ^a^ Exposure to air pollution mixtures alone or exposure to meteorological factors alone；This model adjusted for the covariates.  ^b^ Mixed exposure to air pollution mixtures and meteorological factors; The model was not adjusted for covariates. | | |


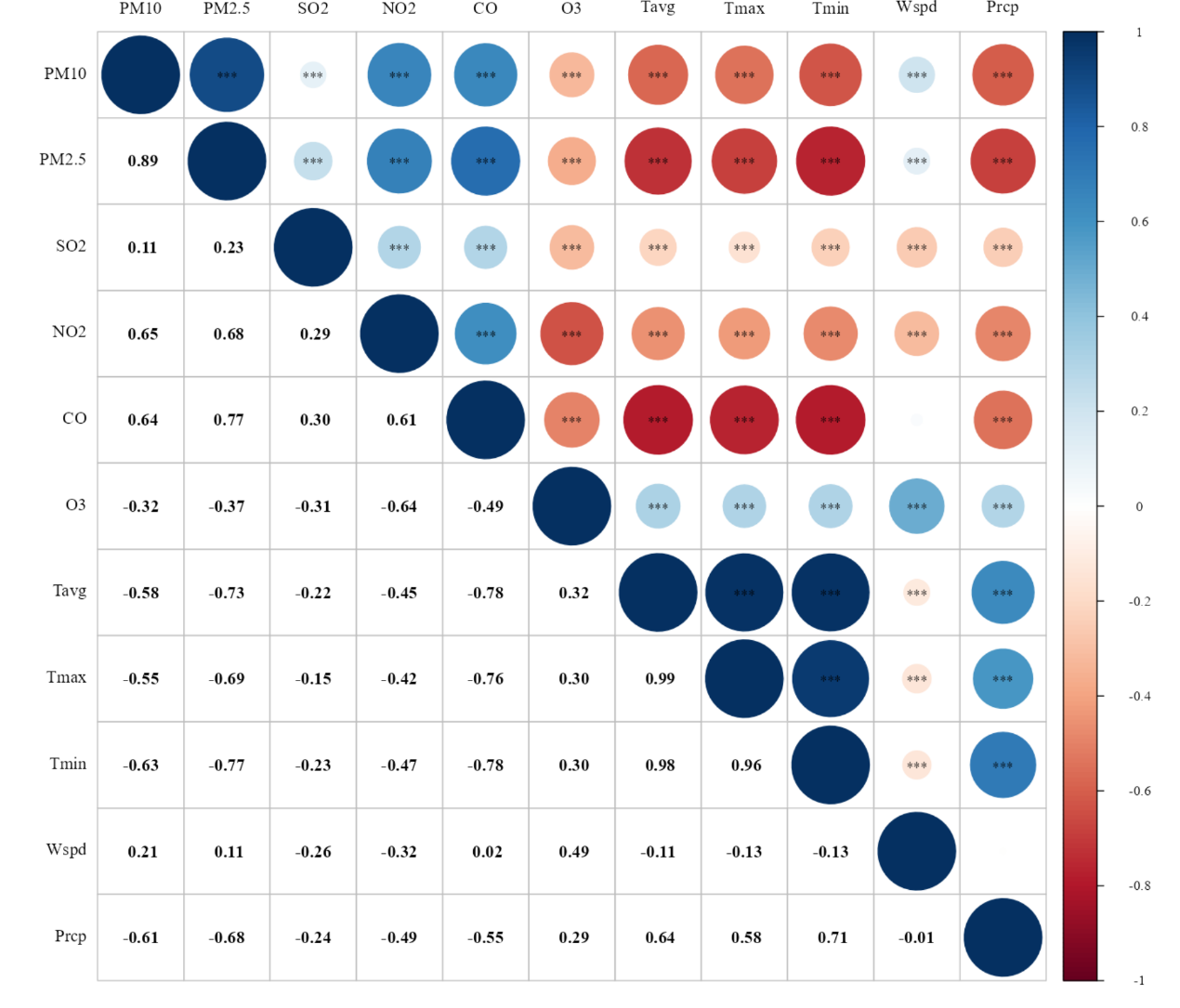


# **Supplementary Figure 1** Correlation between daily air pollutant concentrations and meteorological variables


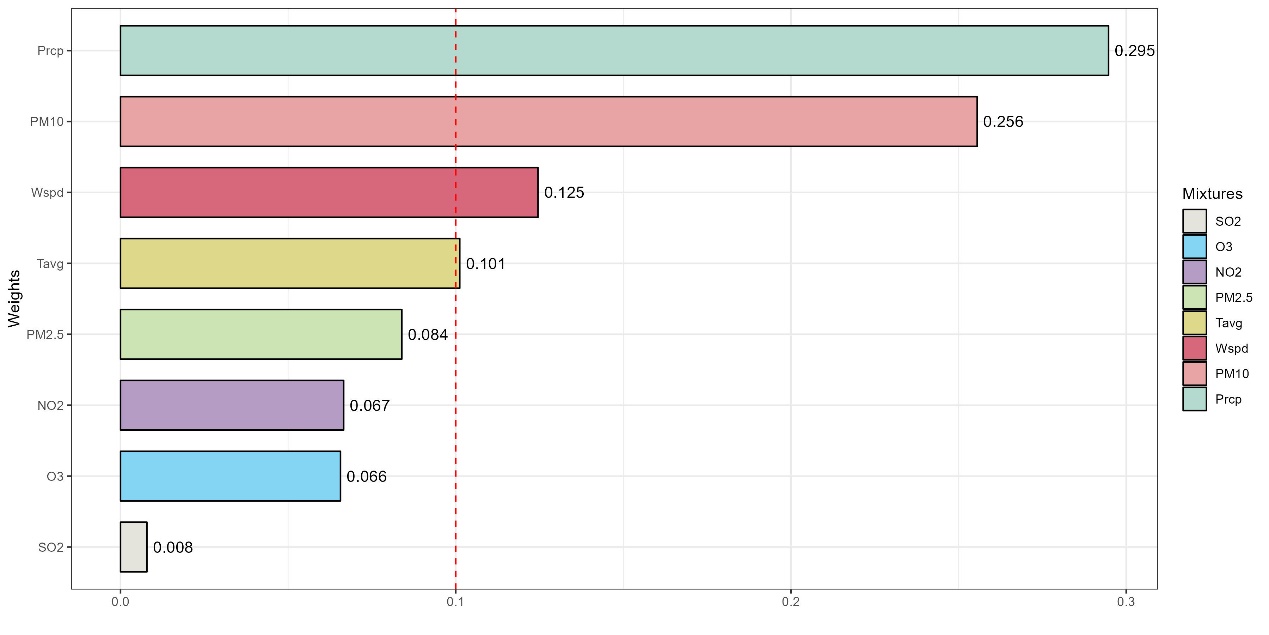


# Supplementary Figure 2 The WQS model weights of air pollution mixtures and meteorological factors on the number of tuberculosis cases in positive direction. The model was adjusted for all covariates.


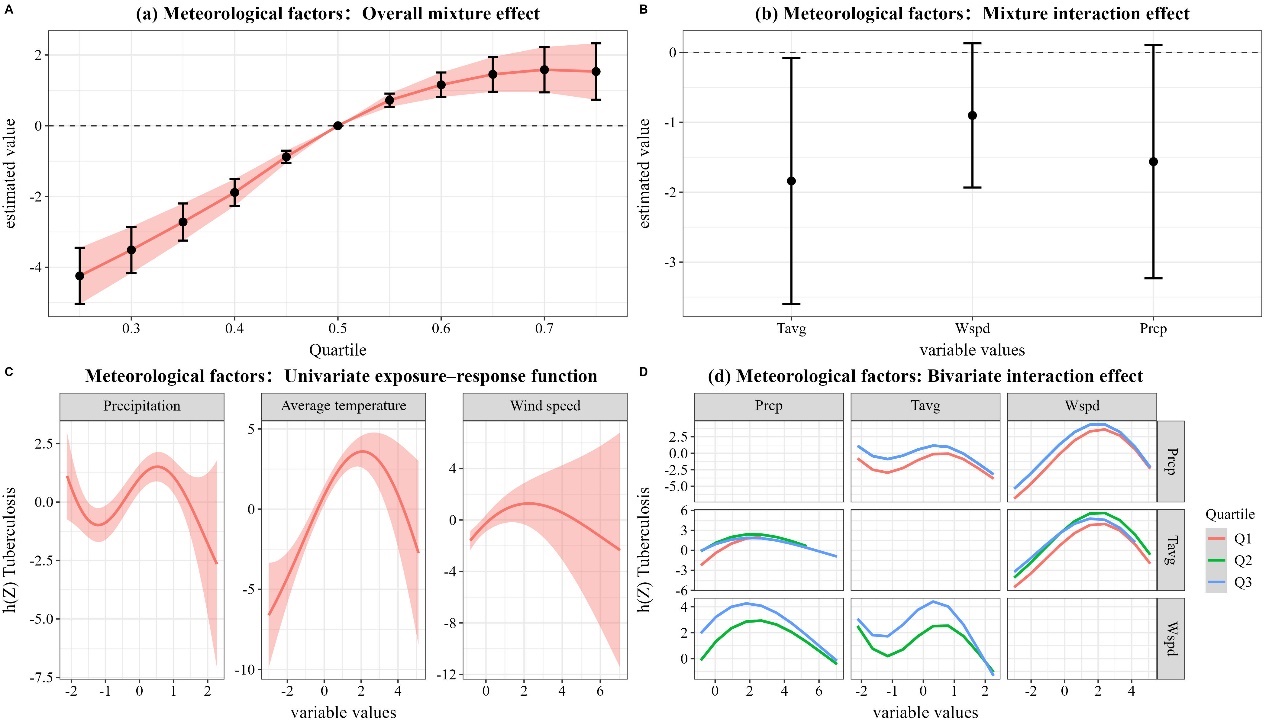


# Supplementary Figure 3 The BKMR model for joint effect analysis of meteorological factors on the number of tuberculosis cases. The model adjusted for all covariates.

**
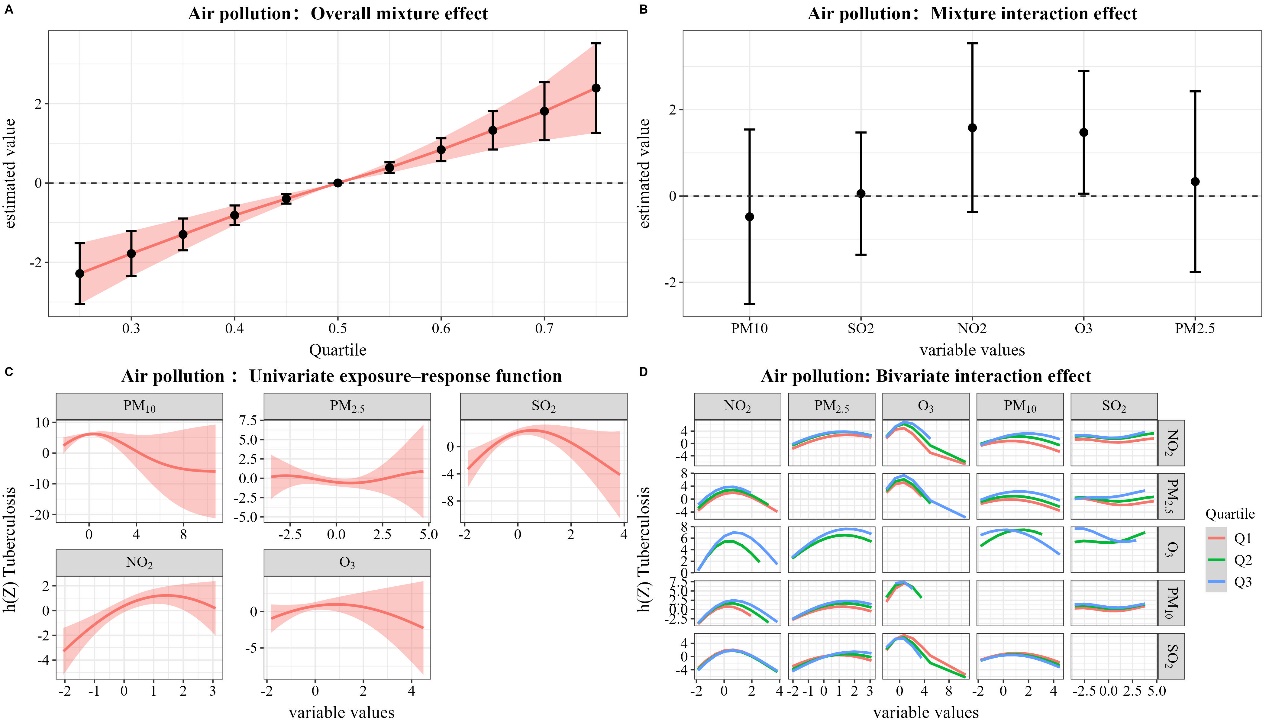
**

# Supplementary Figure 4 The BKMR model for joint effect analysis of air pollutants on the number of tuberculosis cases. The model adjusted for all covariates.


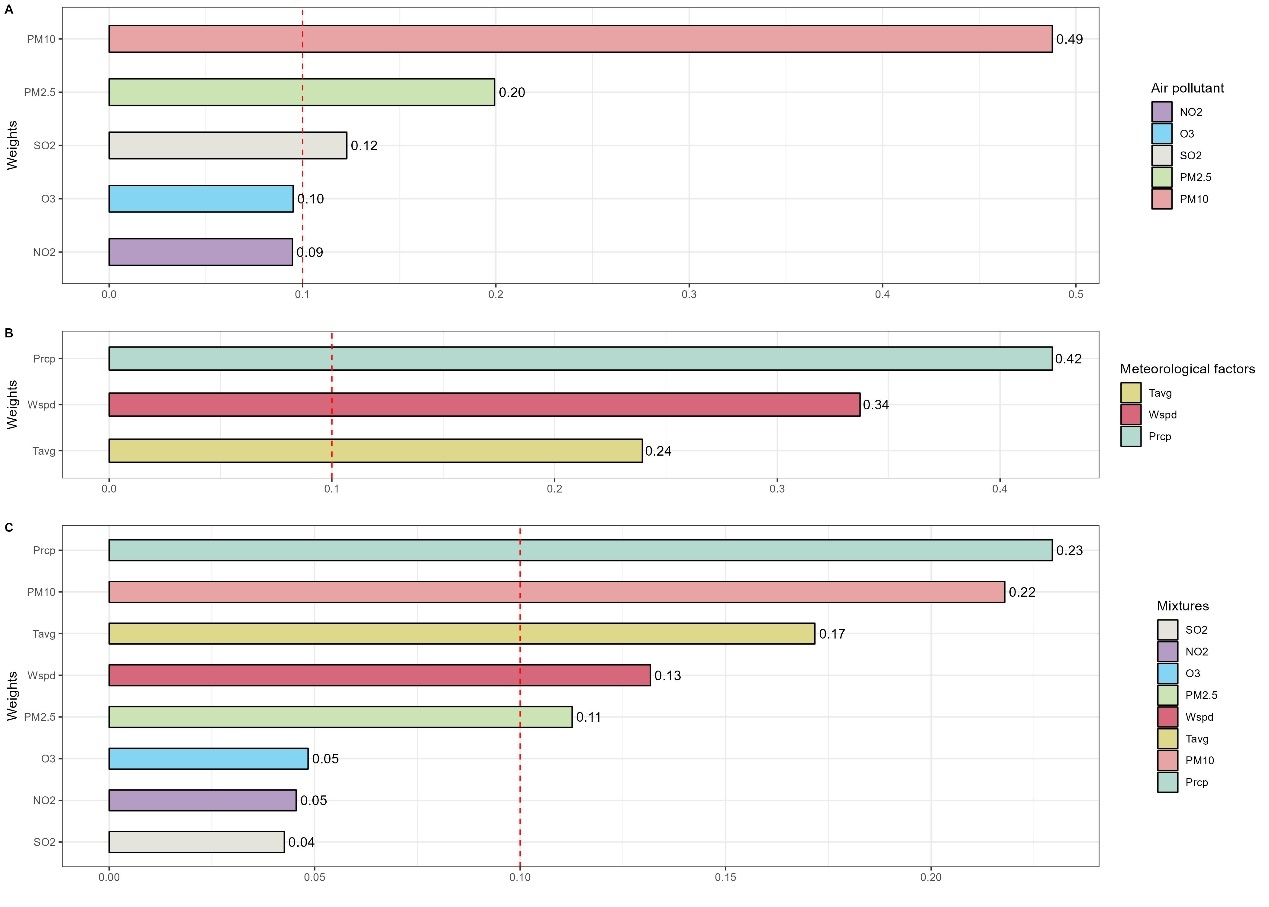


# Supplementary Figure 5 The WQS model weights of the effects of air pollution mixture and meteorological factors on the number of tuberculosis cases in the male subgroup are positive. The model A and B were adjusted for all covariates and the model C was not adjusted for covariates.


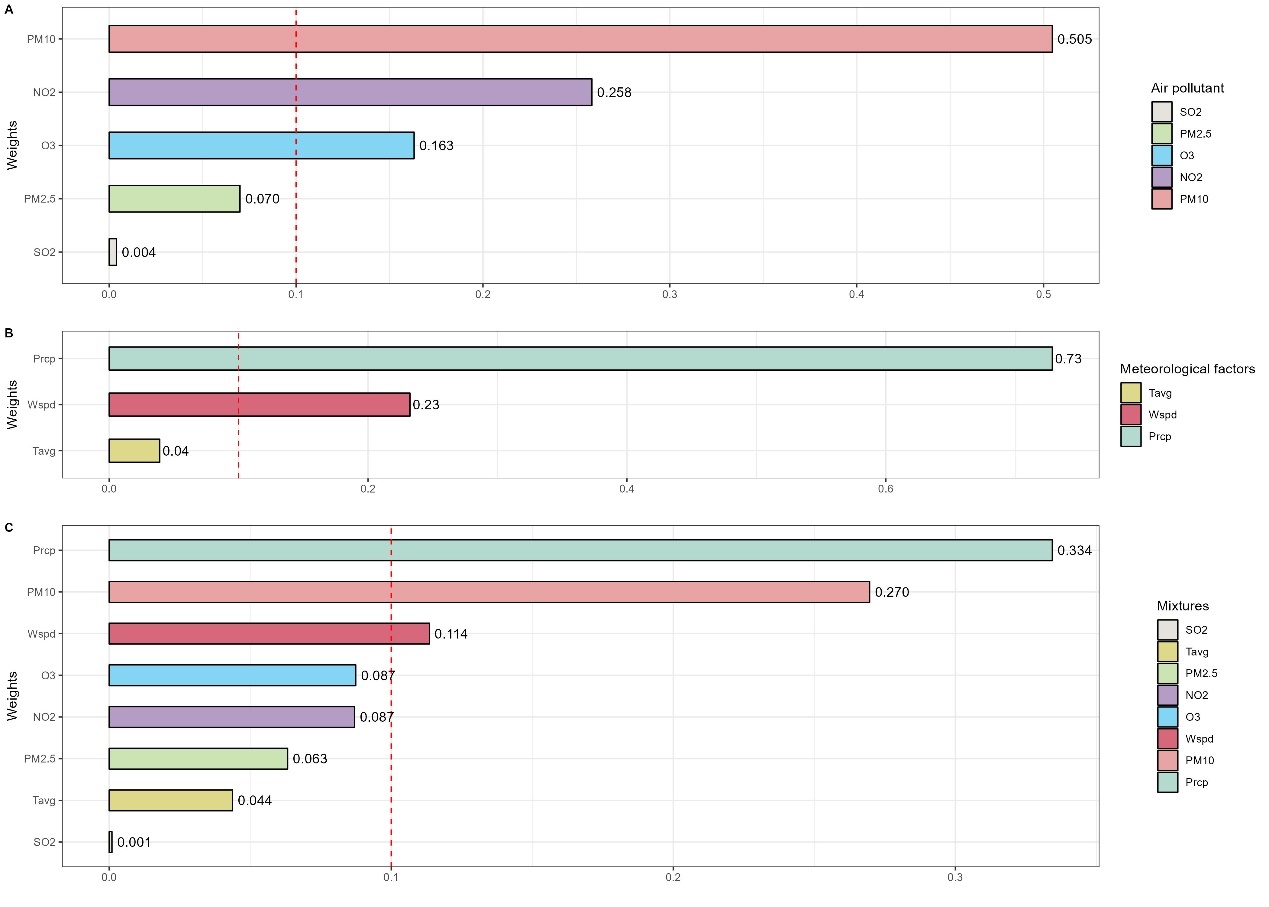


# Supplementary Figure 6 The WQS model weights of the effects of air pollution mixture and meteorological factors on the number of tuberculosis cases in the female subgroup are positive. The model A and B were adjusted for all covariates and the model C was not adjusted for covariates.


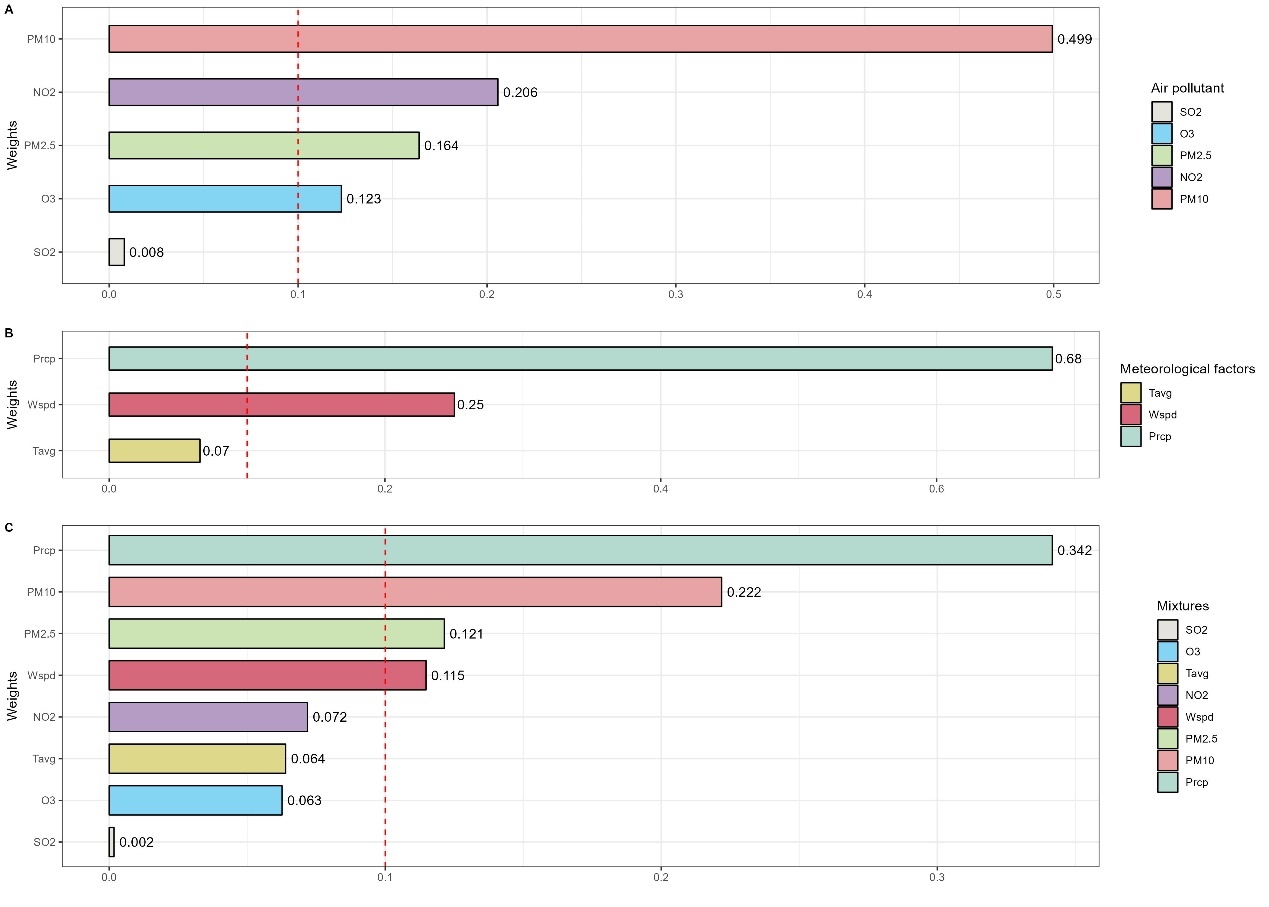


# Supplementary Figure 7 The WQS model weights of the effects of air pollution mixture and meteorological factors on the number of tuberculosis cases in the nomad subgroup are positive. The model A and B were adjusted for all covariates and the model C was not adjusted for covariates.

**
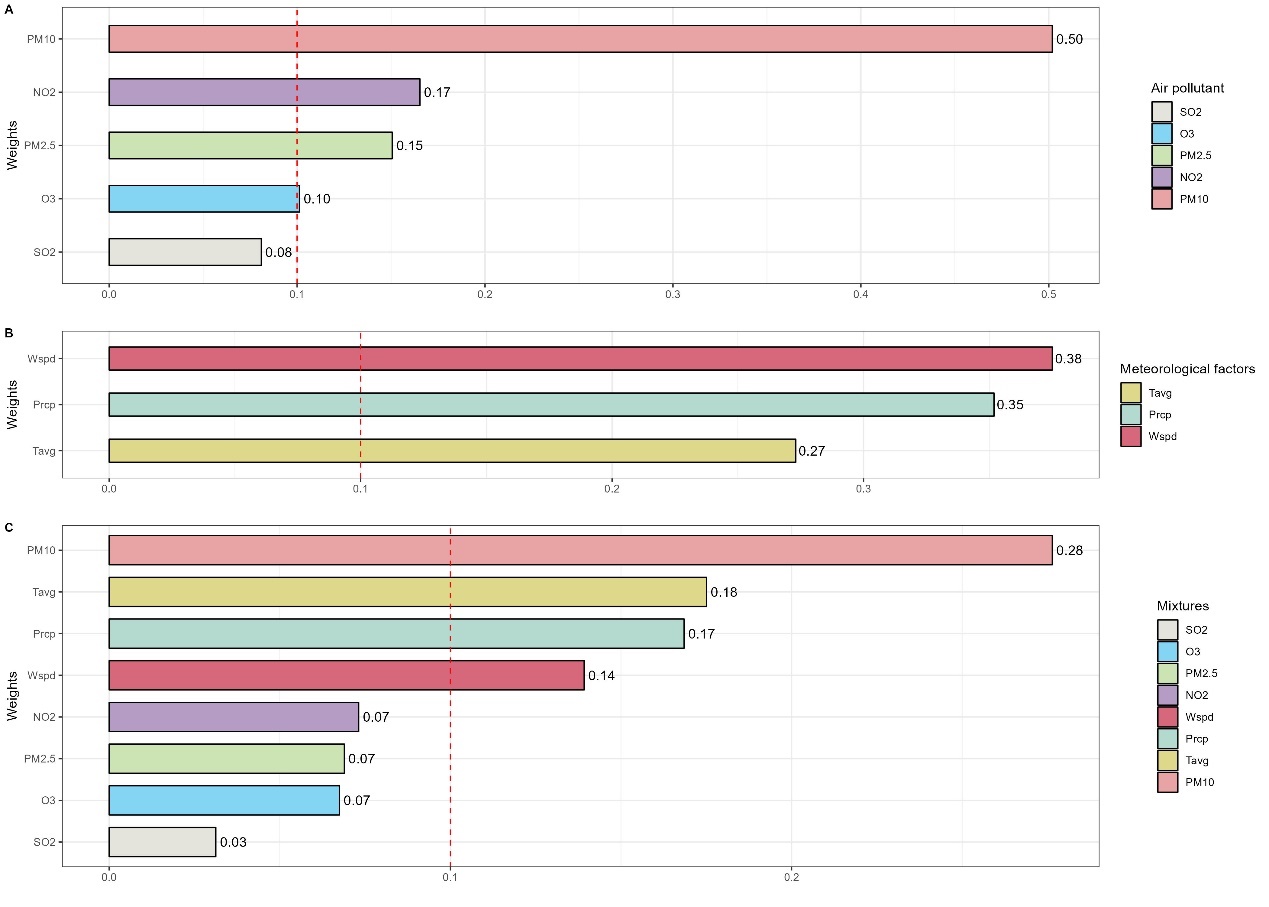
**

# Supplementary Figure 8 The WQS model weights of the effects of air pollution mixture and meteorological factors on the number of tuberculosis cases in the student subgroup are positive. The model A and B were adjusted for all covariates and the model C was not adjusted for covariates.

**
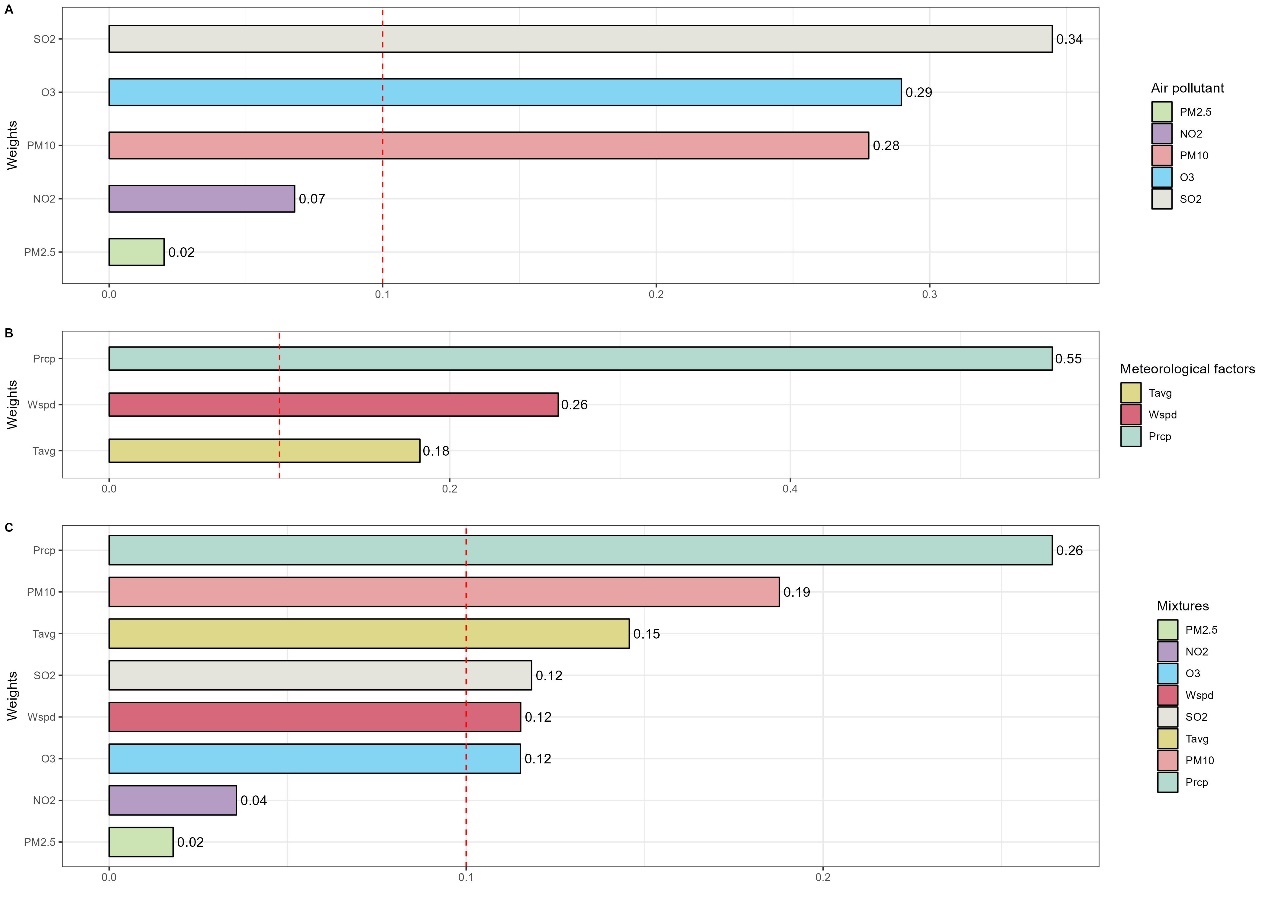
**

# Supplementary Figure 9 The WQS model weights of the effects of air pollution mixture and meteorological factors on the number of tuberculosis cases in the other occupations subgroup are positive. The model A and B were adjusted for all covariates and the model C was not adjusted for covariates.

**
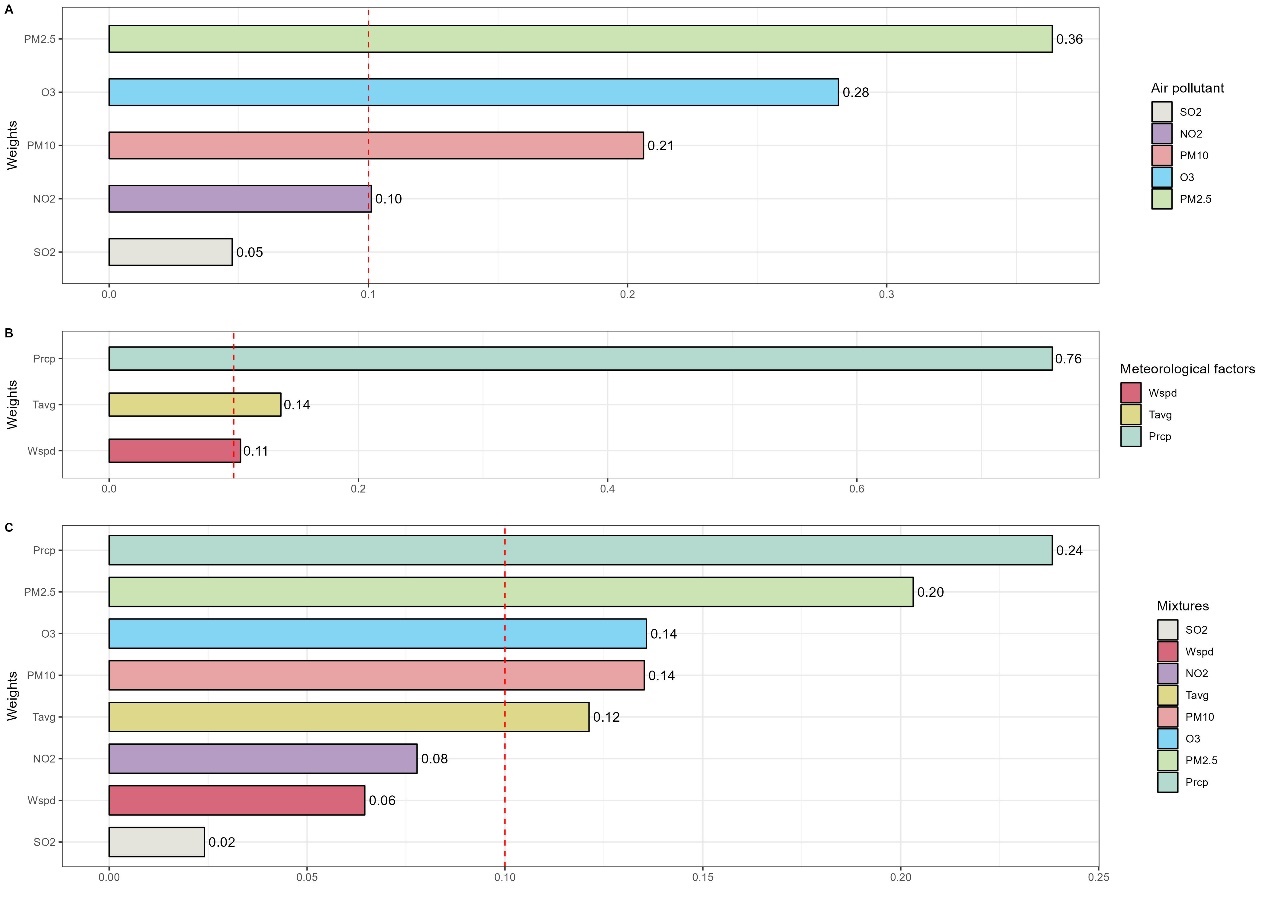
**

# Supplementary Figure 10 The WQS model weights of the effects of air pollution mixture and meteorological factors on the number of tuberculosis cases between year of 2019 and 2021 subgroup are positive. The model A and B were adjusted for all covariates and the model C was not adjusted for covariates.

**
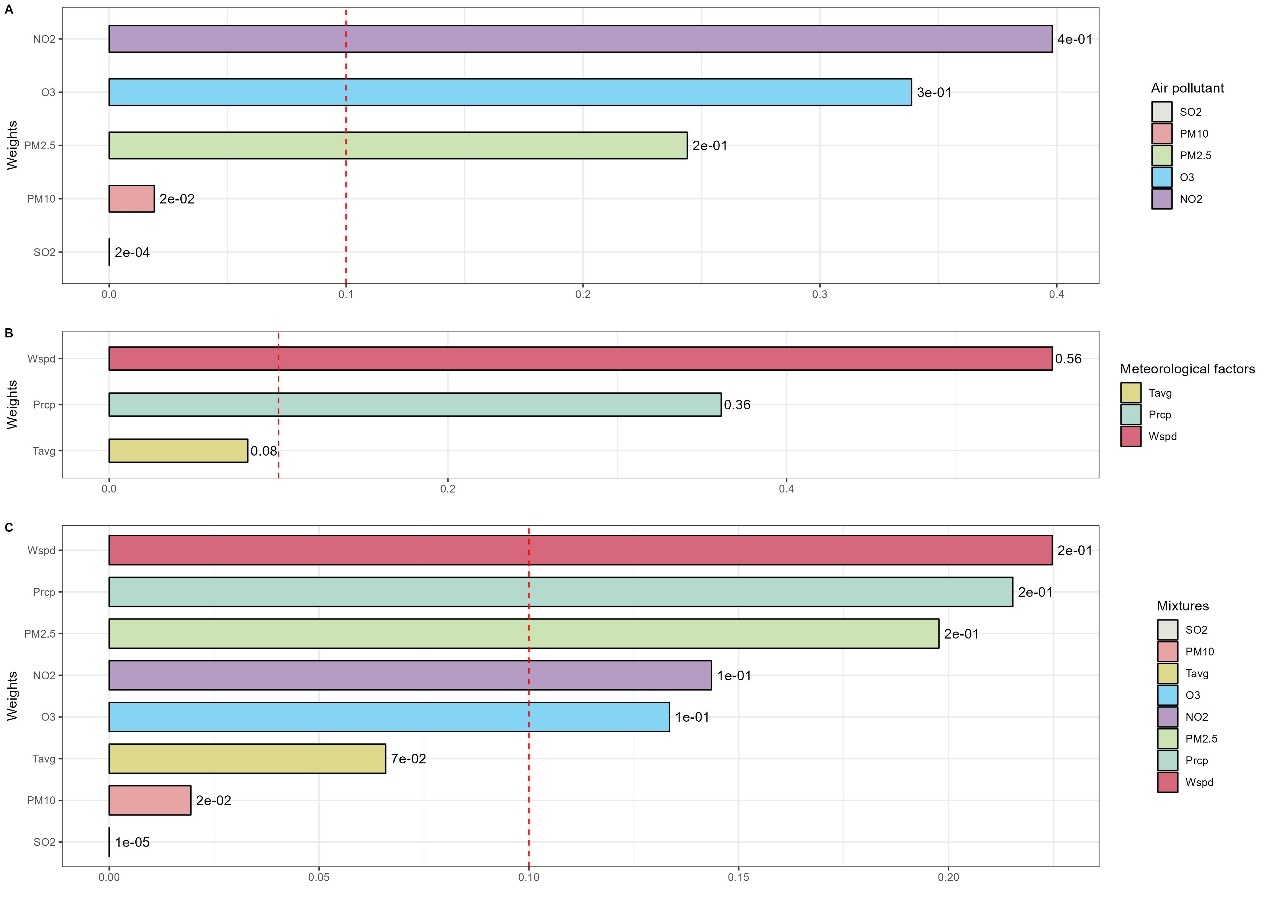
**

# **Supplementary Figure 11** The WQS model weights of the effects of air pollution mixture and meteorological factors on the number of tuberculosis cases between year of 2022 and 2023 subgroup are positive. The model A and B were adjusted for all covariates and the model C was not adjusted for covariates.


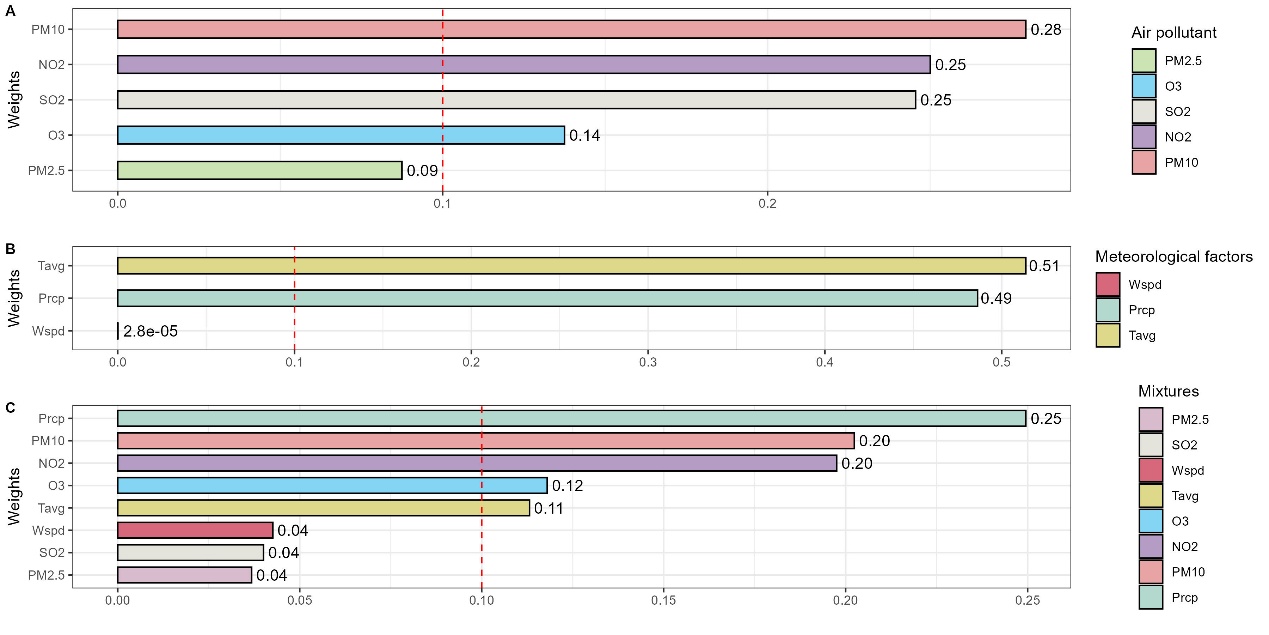


# **Supplementary Figure 12** The WQS model weights of the effects of air pollution mixture and meteorological factors on the number of tuberculosis cases by monthly. The model A and B were adjusted for all covariates and the model C was not adjusted for covariates.


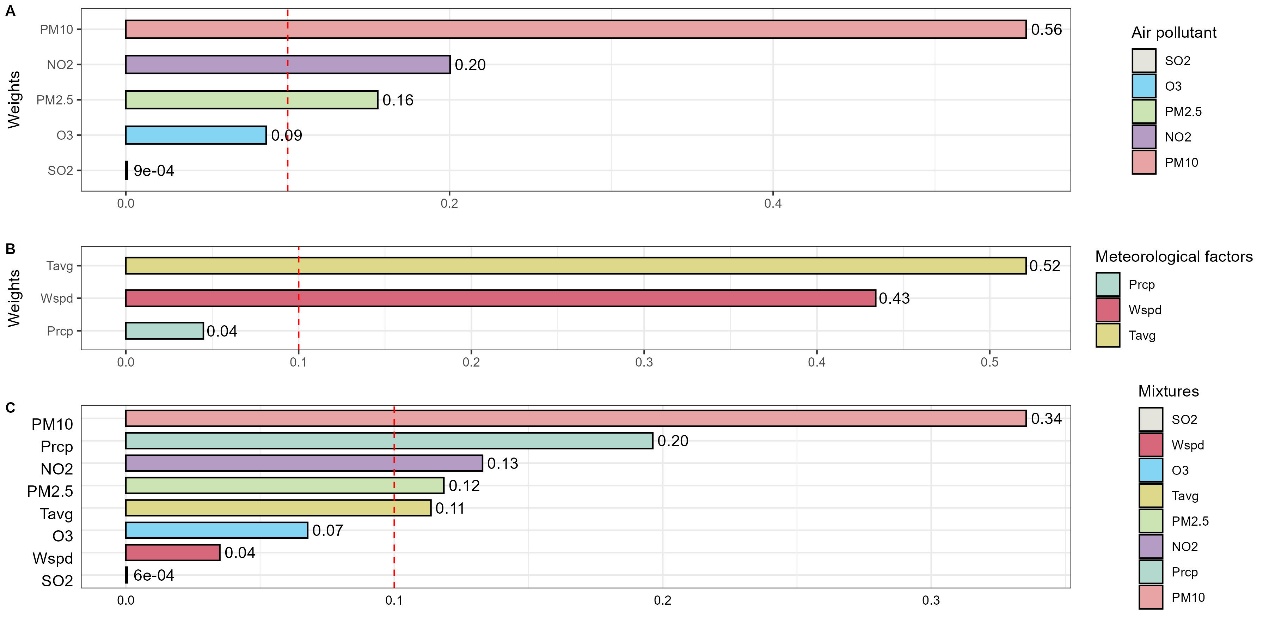


# **Supplementary Figure 13** The WQS model weights of the effects of air pollution mixture and meteorological factors on the number of tuberculosis cases when adjusting for time trends. The model A and B were adjusted for all covariates and the model C was just adjusted for time trends.

**
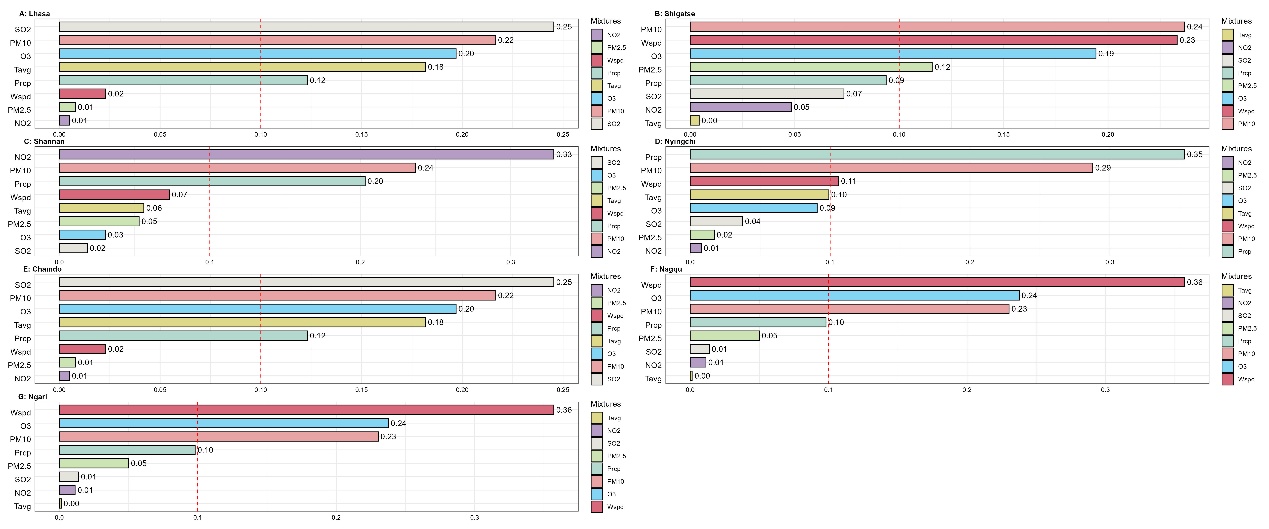
**

# **Supplementary Figure 14** The WQS model weights of air pollution mixtures and meteorological factors on the number of tuberculosis cases in positive direction (With different regions)

**
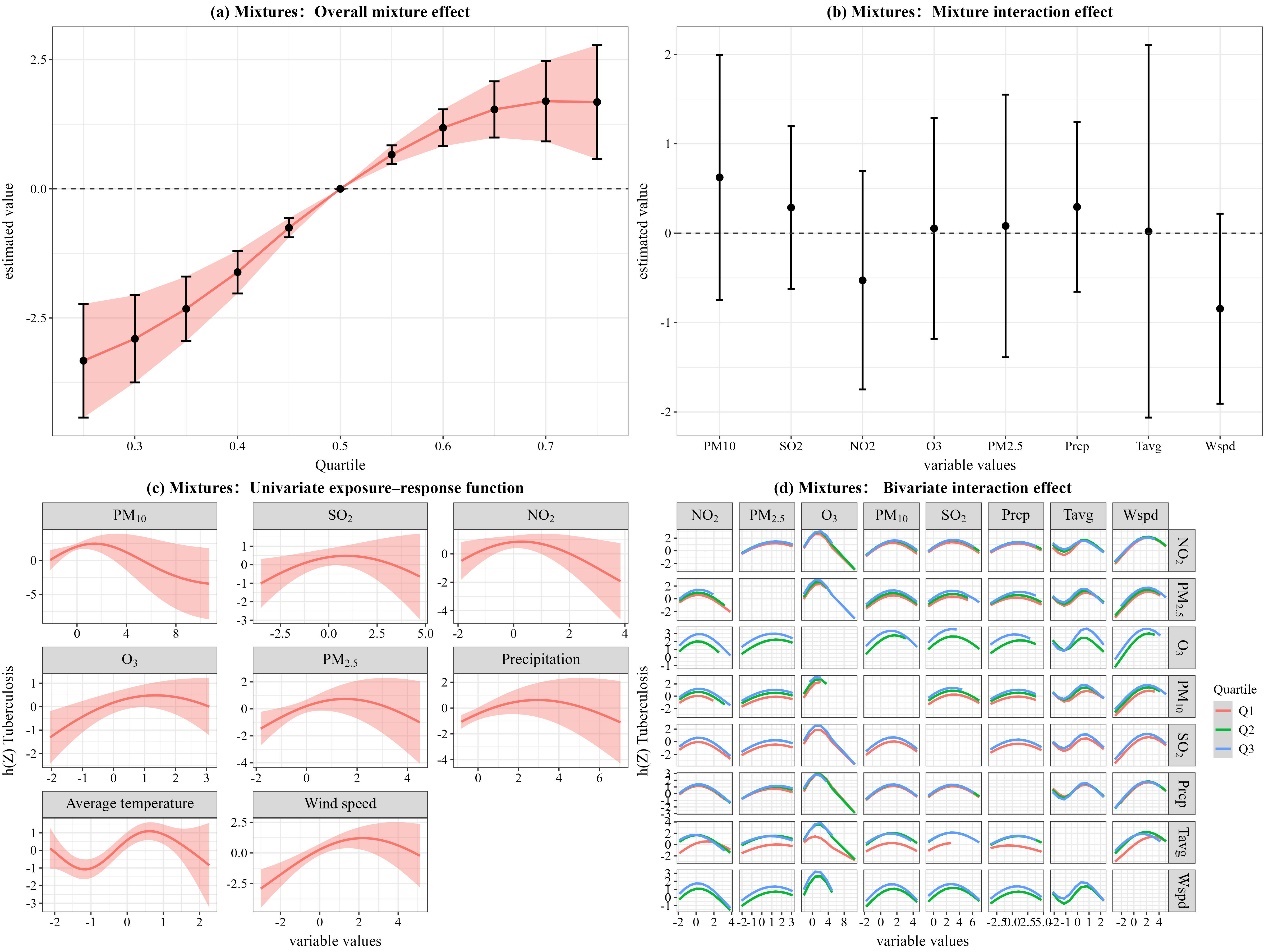
**

# Supplementary Figure 15 The BKMR model for joint effect analysis of air pollutants on the number of tuberculosis cases in the male subgroup. The model was not adjusted for covariates.

**
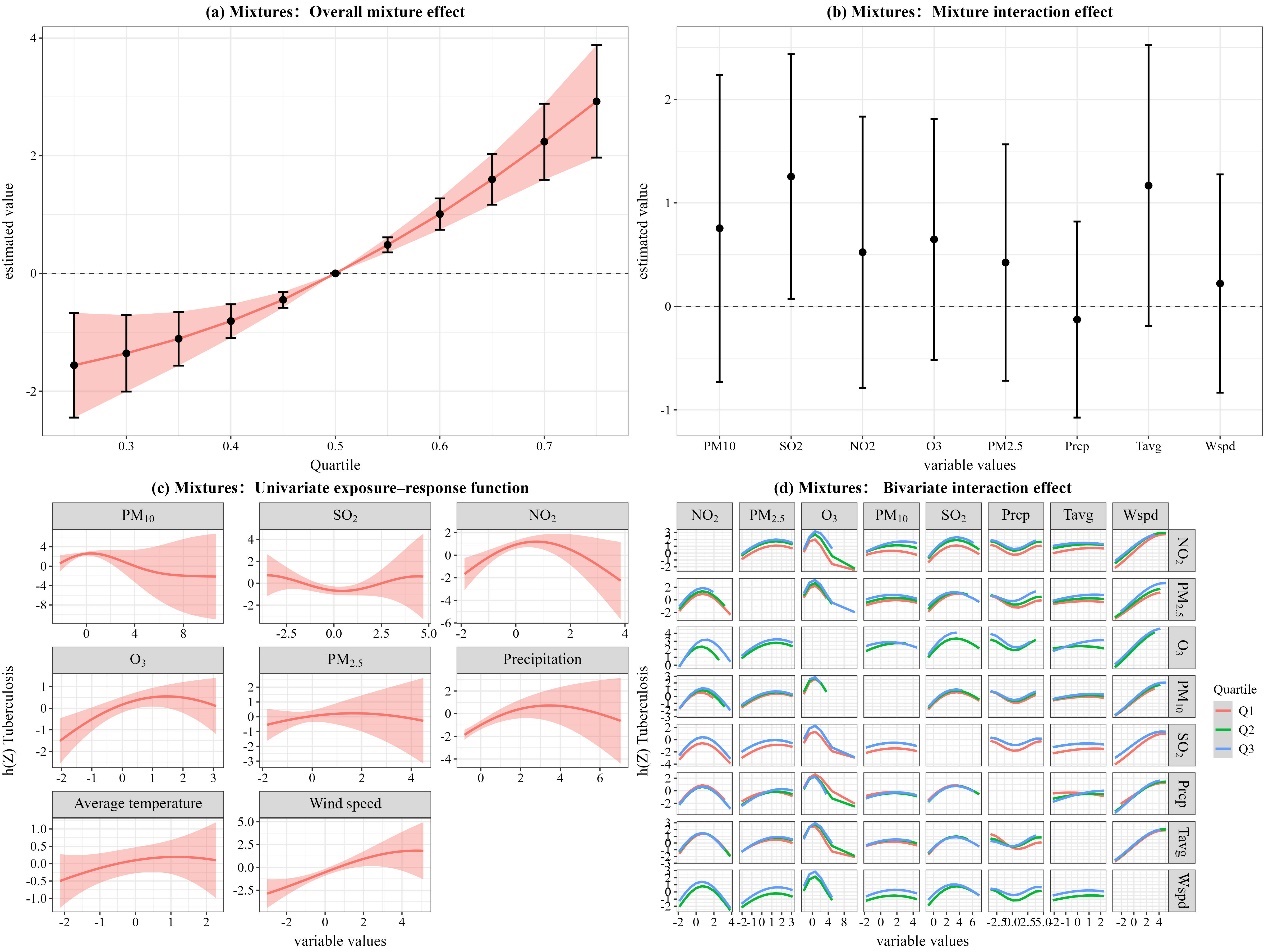
**

# **Supplementary Figure 16** The BKMR model for joint effect analysis of air pollutants on the number of tuberculosis cases in the female subgroup. The model was not adjusted for covariates.

**
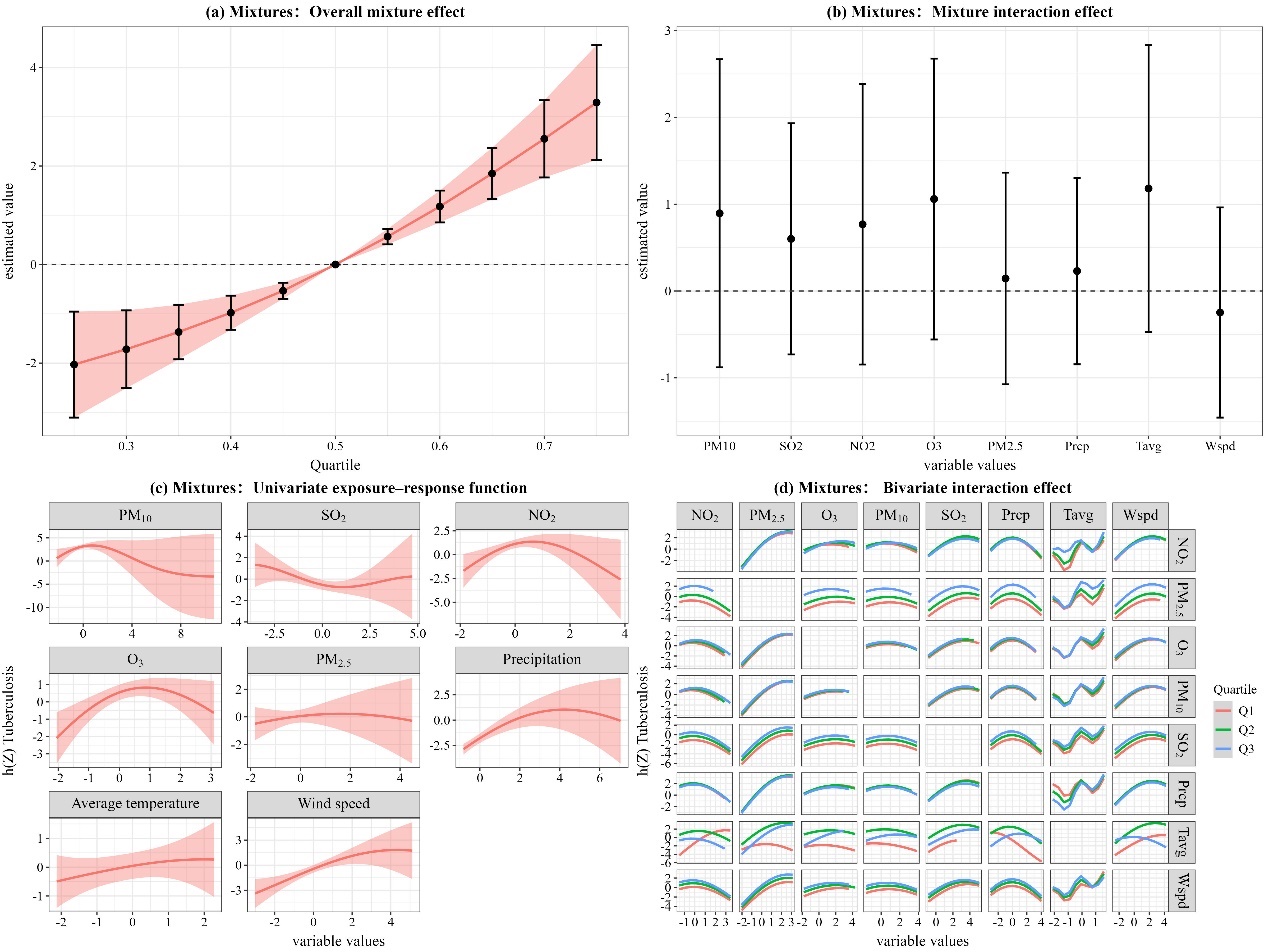
**

# **Supplementary Figure 17** The BKMR model for joint effect analysis of air pollutants on the number of tuberculosis cases in the nomad subgroup. The model was not adjusted for covariates.

**
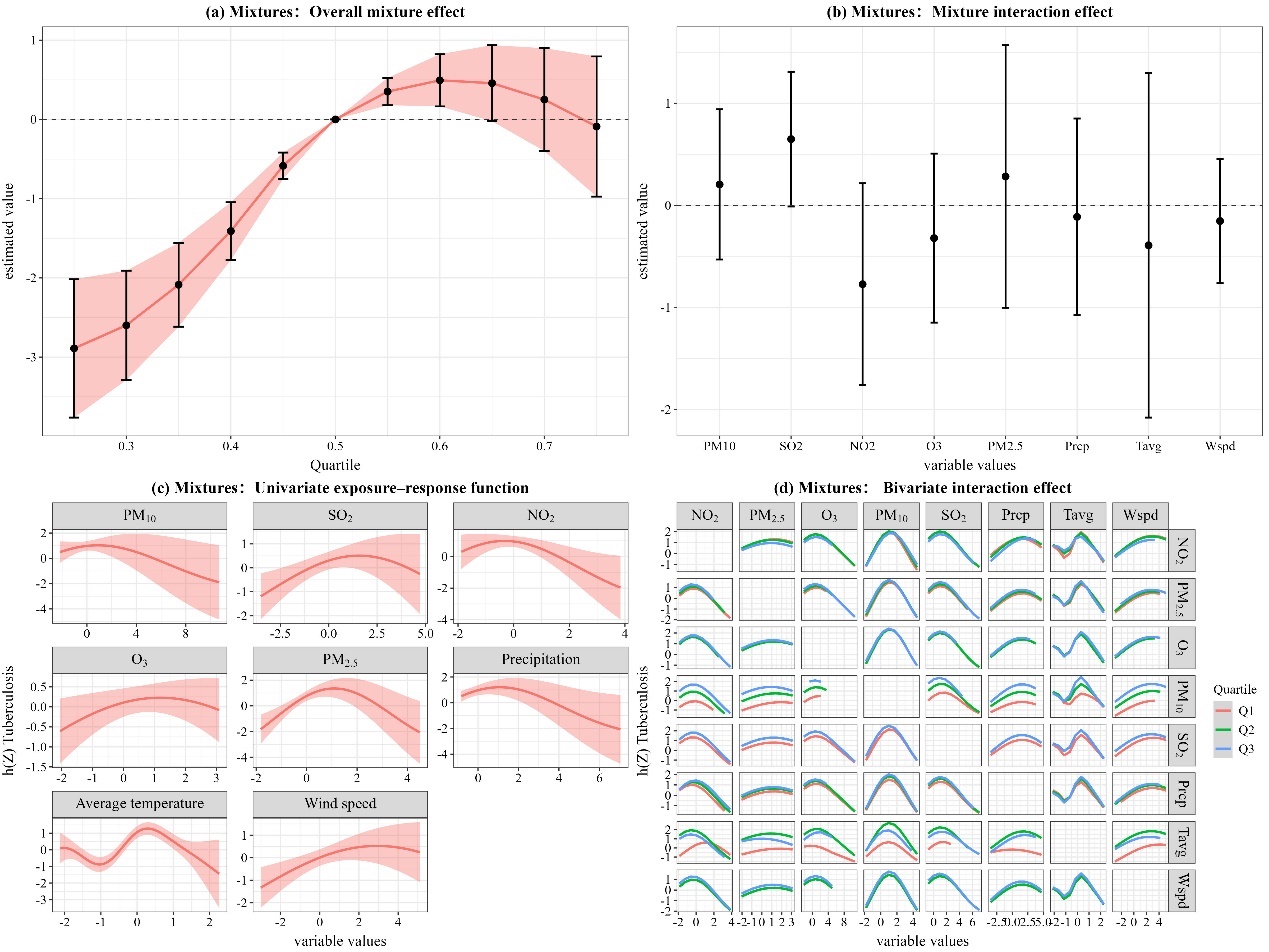
**

# **Supplementary Figure 18** The BKMR model for joint effect analysis of air pollutants on the number of tuberculosis cases in the student subgroup. The model was not adjusted for covariates.

**
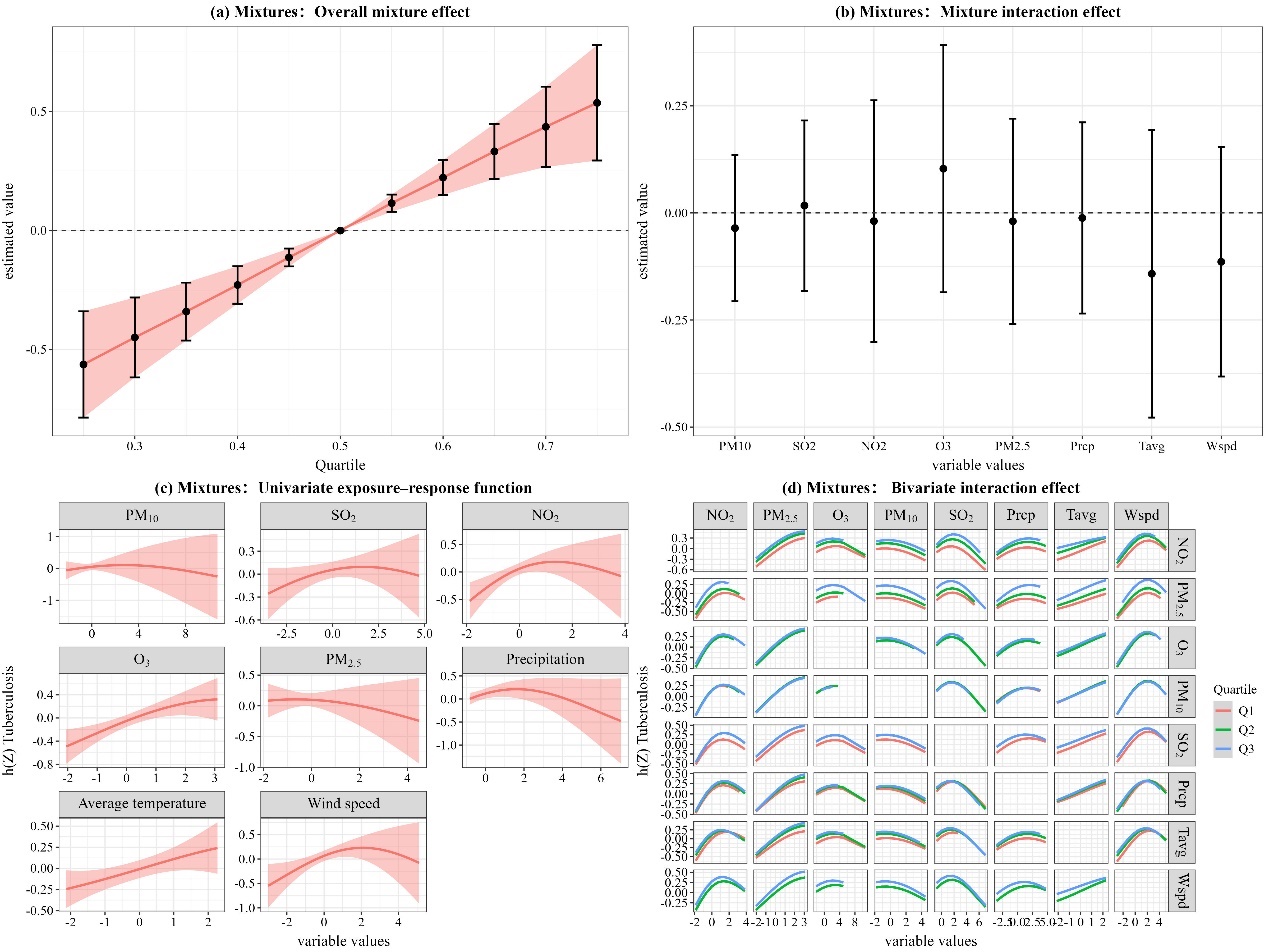
**

# **Supplementary Figure 19** The BKMR model for joint effect analysis of air pollutants on the number of tuberculosis cases in the other occupation subgroup. The model was not adjusted for covariates.

**
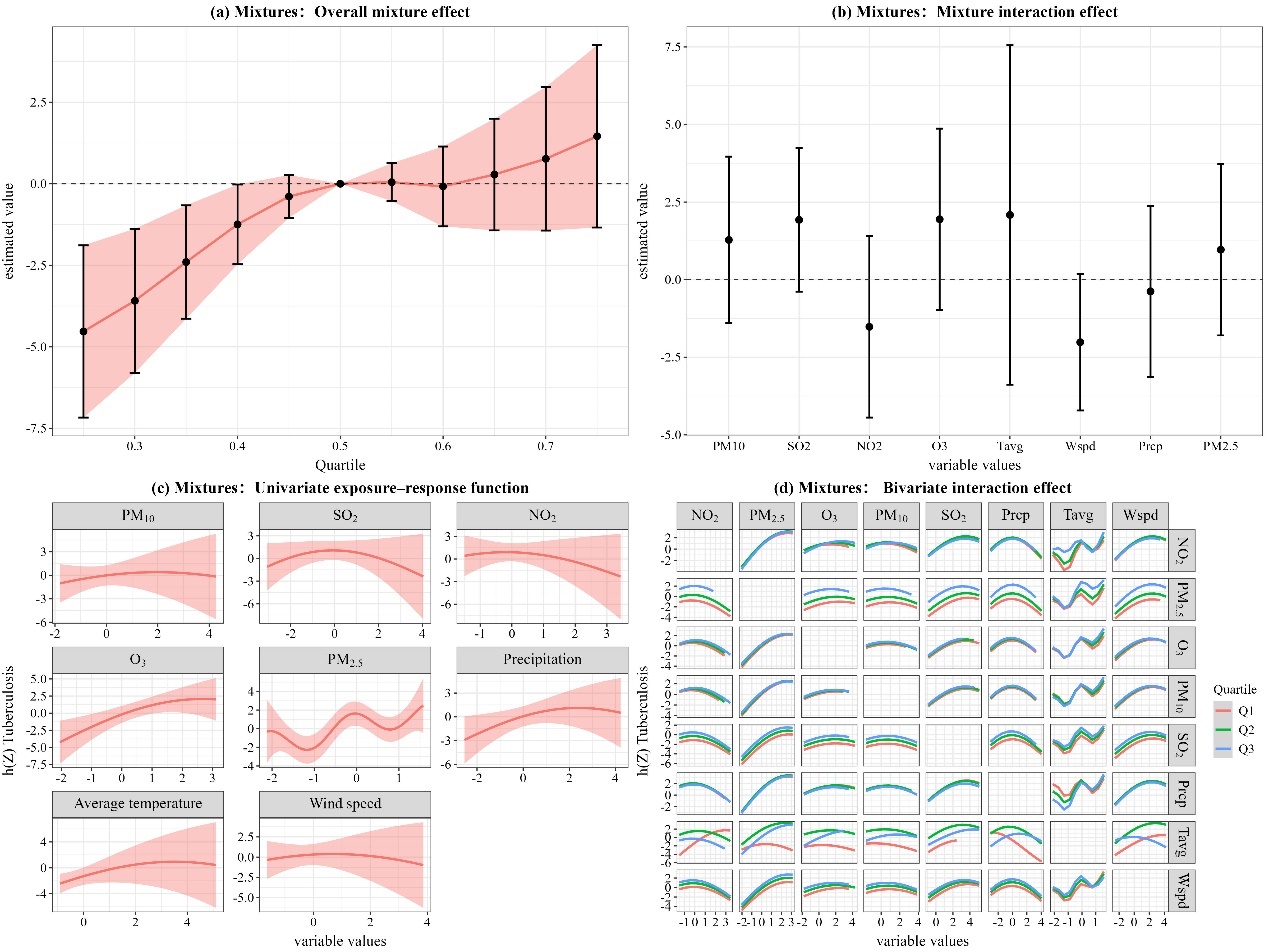
**

# **Supplementary Figure 20** The BKMR model for joint effect analysis of air pollutants on the number of tuberculosis cases between the year of 2019 to 2021. The model was not adjusted for covariates.

**
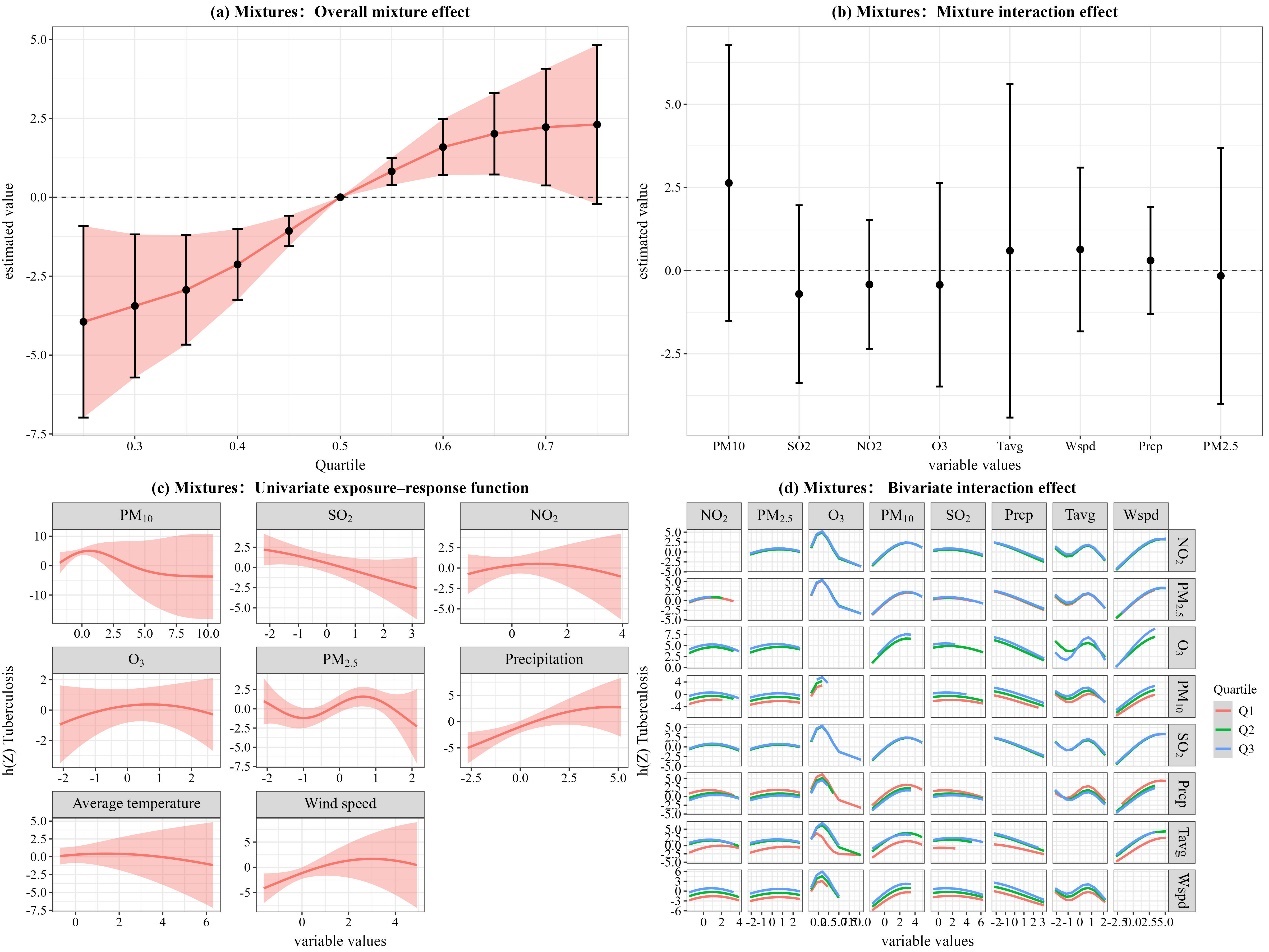
**

# **Supplementary Figure 21** The BKMR model for joint effect analysis of air pollutants on the number of tuberculosis cases between the year of 2022 to 2023. The model was not adjusted for covariates.

**
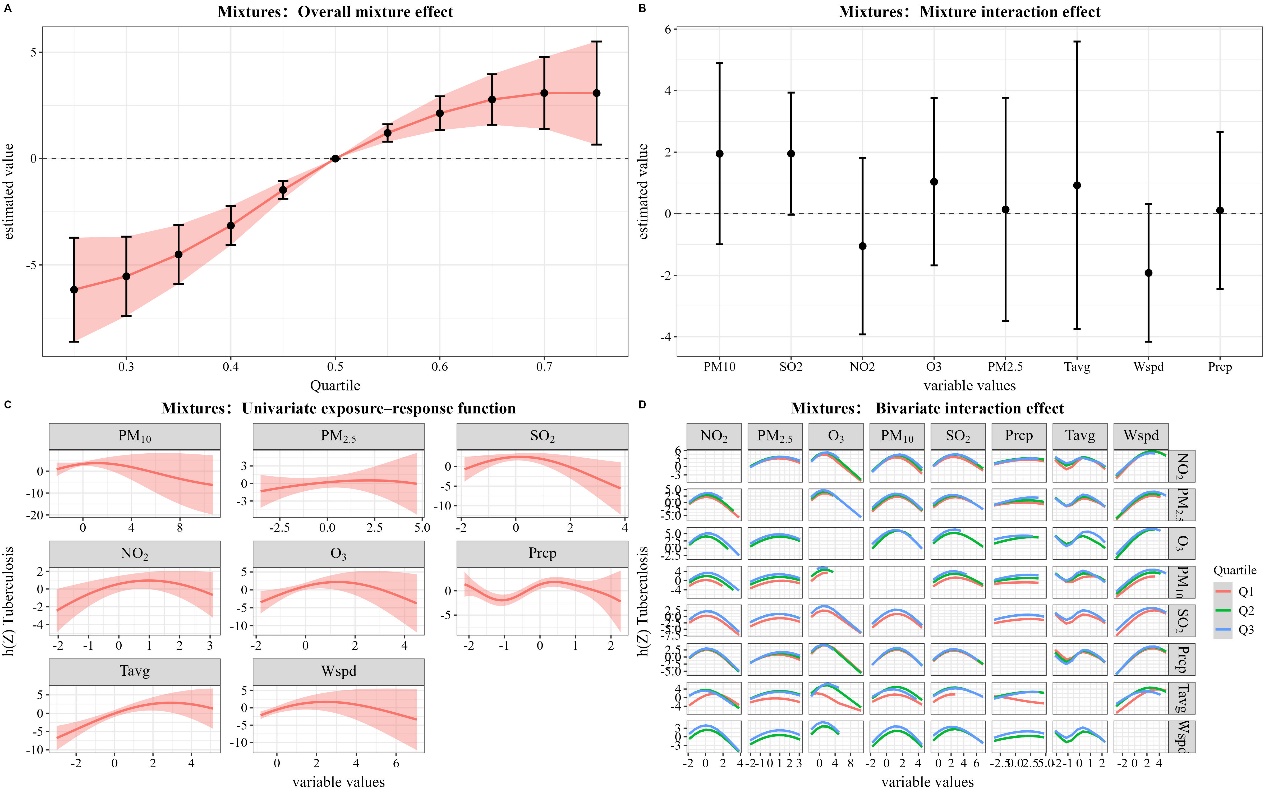
**

# **Supplementary Figure 22** The BKMR model for joint effect analysis of air pollutants on the number of tuberculosis cases by monthly.


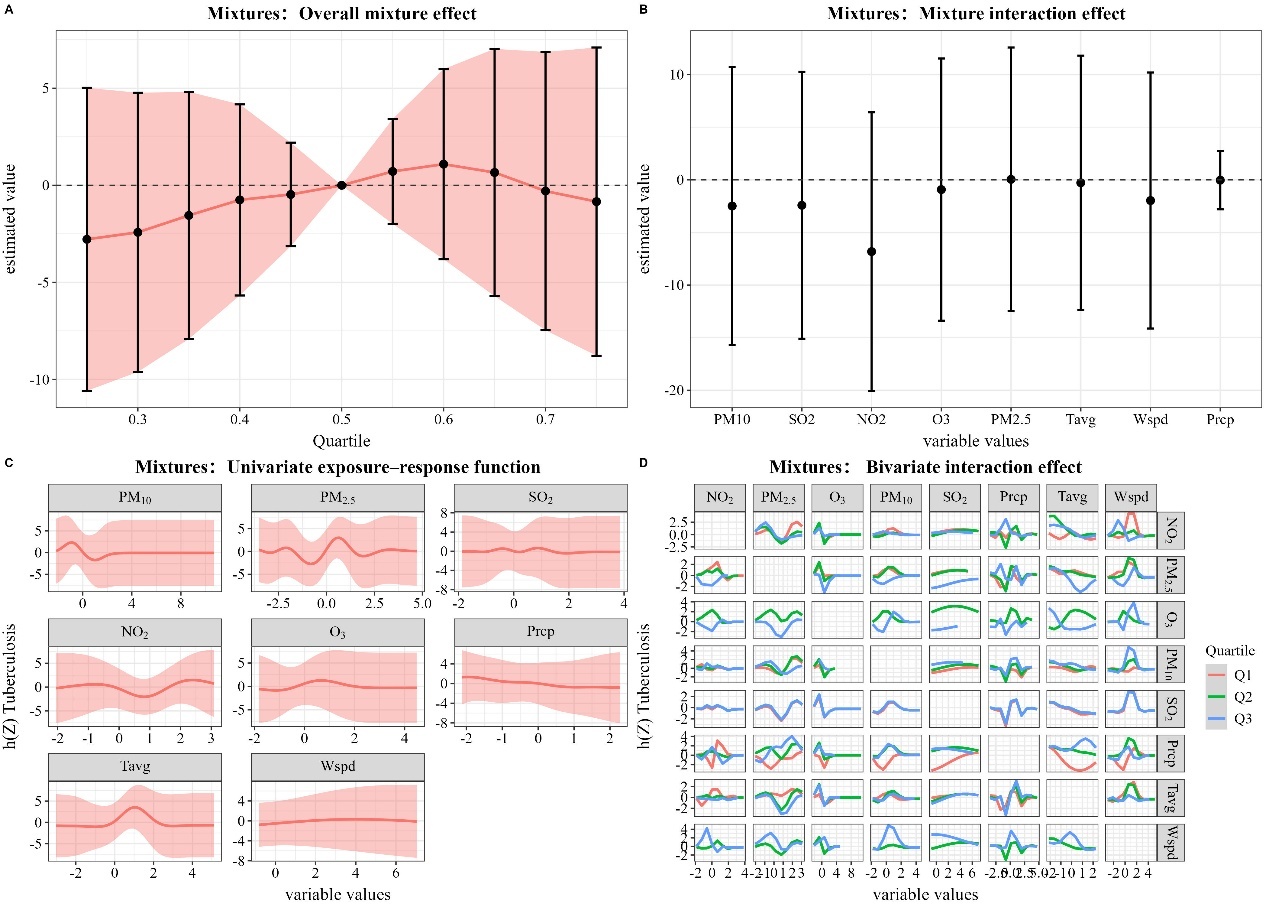


# **Supplementary Figure 23** The BKMR model for joint effect analysis of air pollutants on the number of tuberculosis cases when adjusting for time trends.


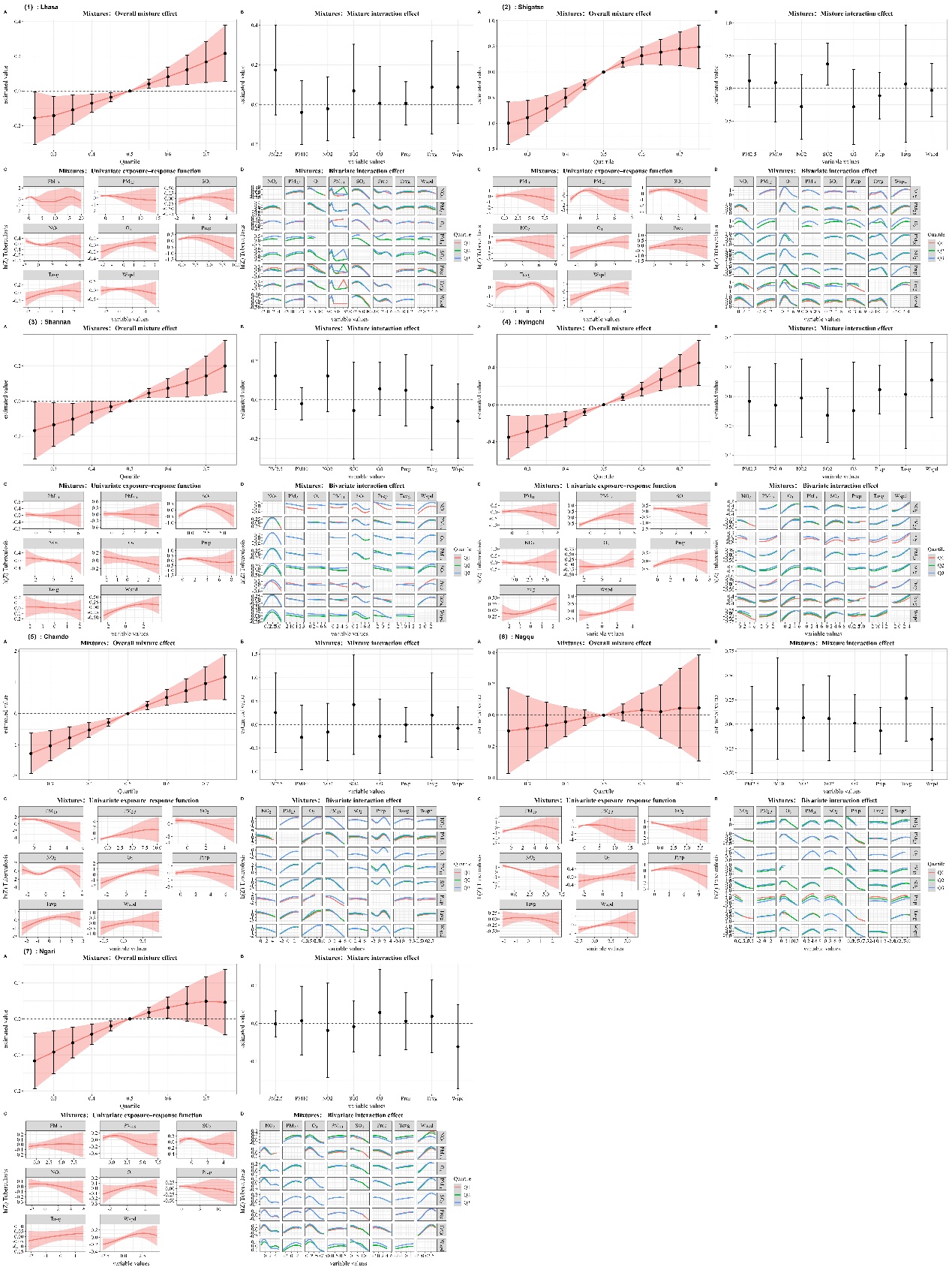


# **Supplementary Figure 24** Overall effect of environment mixtures on the number of tuberculosis cases in BKMR model (With different regions).
